# Integrating genetics and epigenetics in breast cancer: biological insights, experimental, computational methods and therapeutic potential

**DOI:** 10.1186/s12918-015-0211-x

**Published:** 2015-09-21

**Authors:** Claudia Cava, Gloria Bertoli, Isabella Castiglioni

**Affiliations:** Institute of Molecular Bioimaging and Physiology (IBFM), National Research Council (CNR), Milan, Italy

## Abstract

**Background:**

Development of human cancer can proceed through the accumulation of different genetic changes affecting the structure and function of the genome. Combined analyses of molecular data at multiple levels, such as DNA copy-number alteration, mRNA and miRNA expression, can clarify biological functions and pathways deregulated in cancer. The integrative methods that are used to investigate these data involve different fields, including biology, bioinformatics, and statistics.

**Results:**

These methodologies are presented in this review, and their implementation in breast cancer is discussed with a focus on integration strategies. We report current applications, recent studies and interesting results leading to the identification of candidate biomarkers for diagnosis, prognosis, and therapy in breast cancer by using both individual and combined analyses.

**Conclusion:**

This review presents a state of art of the role of different technologies in breast cancer based on the integration of genetics and epigenetics, and shares some issues related to the new opportunities and challenges offered by the application of such integrative approaches.

## Introduction

Breast Cancer (BC) is the most common cancer in women and the second most common cause of cancer mortality among females [1]. Classification of BC is currently based on histological types and molecular subtypes in order to reflect the hormone-responsiveness of the tumour. The three most common histological types include invasive ductal carcinoma, ductal carcinoma in situ and invasive lobular carcinoma. The molecular subtypes of BC, which are based on the presence or absence of estrogen receptors (ER), progesterone receptors (PR), and human epidermal growth factor receptor-2 (HER2), include luminal A (ER+ and/or PR+; HER2–), luminal B (ER+ and/or PR+; HER2+), basal-like (ER–, PR–, and HER2–), and HER2-enriched (ER–, PR–, and HER2+) subtypes [2, 3]. This classification reflects the BC heterogeneity and the complexity of diagnosis, prognosis, and treatment of BC.

High-throughput approaches allow today a tumour to be investigated at multiple levels: (i) DNA with copy number alteration (CNA), ii) epigenetic alterations, specifically, DNA methylation, histone modifications and microRNA (miRNA) expression level alterations, and (iii) mRNA, with gene expression (GE) de-regulation. These high-throughput approaches redefined the different types of BC in terms of classification, showing the presence of only two BC profiles with different prognosis [4–6].

Development of human cancer can proceed through the accumulation of genetic and epigenetic changes affecting the structure and function of the genome. Several studies have reported that the epigenetic silencing of one allele may act in concert with an inactivating genetic alteration in the opposite allele, thus resulting in total allelic loss of the gene [7, 8]. Birgisdottir et al. [9] have reported hypermethylation and deletion of the *BRCA1* promoter and suggested Knudson's two 'hits' in sporadic BC [9]. Li et al. [10] were focused on the expression of *beclin 1* mRNA and they demonstrated that loss of heterozygosity and aberrant DNA methylation might be the possible reasons of the decreased expression of *beclin 1* in the BC. In BC, a biallelic inactivation of the FHIT gene could be a consequence of epigenetic inactivation of both parental alleles, or epigenetic modification of one allele and deletion of the remaining allele [11].

In 2006, Feinberg et al. suggested that epigenetics and genetics should be combined or integrated in order to achieve better understanding of cancer [12]. A systems biology approach has been employed to explore the functional relationships among multidimensional “omics” technologies. This approach has been demonstrated to be important for addressing a patient to the optimal treatment in a personalized way, in order to improve the efficacy of the treatment for that patient [13].

This review refers to current studies of genetic and epigenetic changes associated with BC, focusing in particular on the processes controlled by CNA, epigenetic alterations (DNA methylation, histone modifications and miRNAs), and GE. Several approaches combining genetic and epigenetic data, in particular regarding CNA and miRNA deregulation, have been considered with the final purpose to identify new biomarkers for BC diagnosis and prognosis suitable to be translated into a clinical environment. Furthermore, experimental and computation methods used for the study and the analysis of these biomarkers are presented. We also discuss the biological insights and clinical impact from such analyses as well as the future challenges of these combination approaches.

### Copy number alterations in BC

#### Biological insights

CNAs are alterations of the DNA of a genome that result in a cell having an abnormal number of copies of one or more sections of the DNA. They have been identified as causes of cancer diseases and developmental abnormalities (e.g. [14]). Changes in DNA copy number (CN) can occur in specific genes or involve whole chromosomes, usually genomic regions between 1kbp and 1Mbp in length [14].

**Fig. 1:**
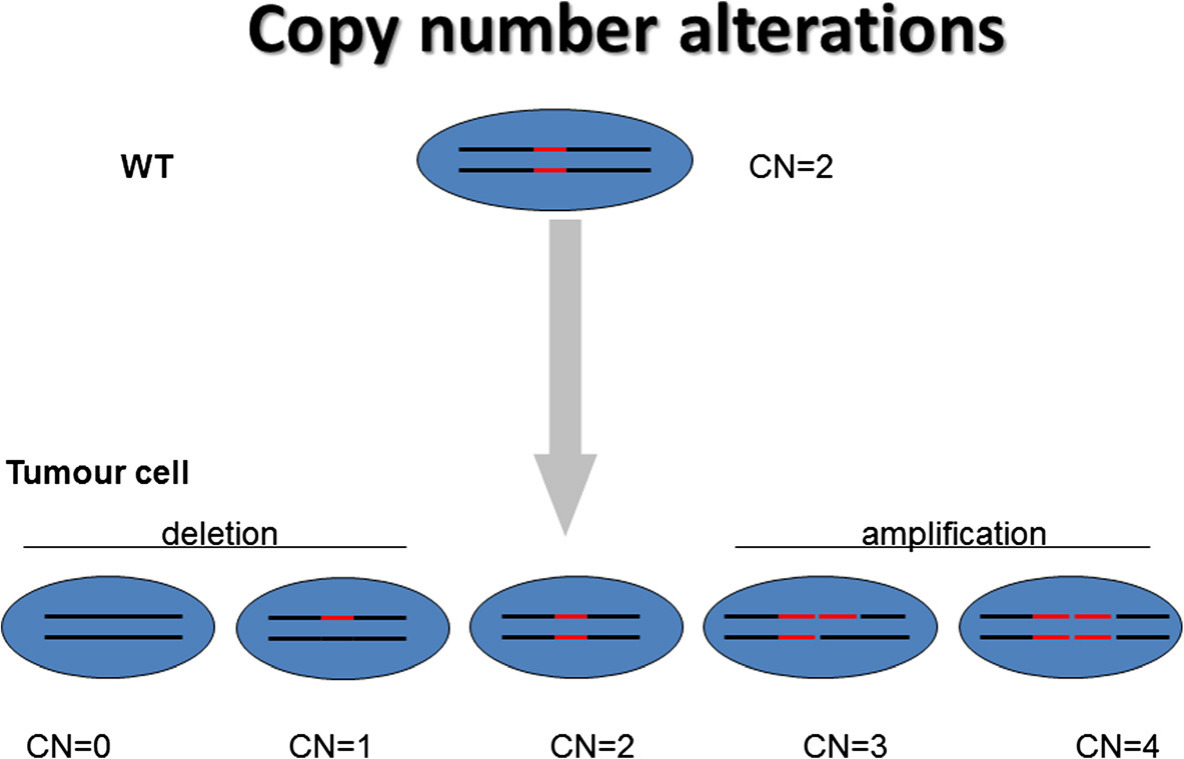
Copy Number alterations. WT cell, since diploid organisms, carry two copies of each gene (red segments). Deletions in tumour cells lead to no copy (CN = 0) or one copy (CN = 1) of this section of DNA, rather than two copies (CN = 2). Amplifications in tumour cells lead to three (CN = 3) or more copies (CN = 4) of DNA section

Figure 1 shows an example of a wild type (WT) cell with two copies of DNA segments that suffer of alterations in tumour cells bringing deletions (CN = 0; CN = 1) or amplifications (CN = 3; CN = 4) of the DNA section.

The ability of cancer cells to accumulate genetic alterations is crucial for the development of cancer in order to inactivate tumour suppressor genes (TSGs) and activate oncogenes (OGs).

In BC, several genetic alterations have been found.

Frequent CN deletions between axillary lymph node metastasis and BC primary tumours were revealed, including aberrations at 6q15-16, containing the gene *PNRC1* (a putative tumour suppressor) [15]. Amplification and overexpression of the *HER2* (HER2/neu, *ERBB2*) oncogene on chromosome 17q12 has been observed in 15–25 % of invasive BC [16]. *HER2*-amplified (HER2+) has been associated with poor prognosis in BC [17], amplification of the *HER2* gene leading to *HER2* protein levels 10–100 times greater than normal levels [18].

EGFR amplification has been frequently associated with indices of poor prognosis in BC patients, such as large tumour size, high histological grade, high proliferative index, HER2 negative, upregulation of PR [19], and negative ER status [20].

In the same region of *HER2* (17q12–21) other genes have been found co-amplified or deleted, e.g. topoisomerase (*TOP2A*) [21]. Different studies observed the possibility of guiding therapy based on *TOP2A* status [22, 23].

A recent study has shown alterations of *PIK3CA* and *MET* in BC [24]. High CN of *PIK3CA* and *MET* was associated to a poor prognosis, and these alterations occur often in triple receptor negative BC [24]. Alterations were also found at 9q31.3-33.1, where the genes *DBC1* and *DEC1* (regulators of apoptosis) are located [15].

OGs activation by genomic amplification occurs in the members of different oncogene families, e.g. *MYC* and *CCND. MYC* is a key regulator of cell growth, proliferation, metabolism, differentiation, and apoptosis [25]. This oncogene is located on chromosome 8q24, and several mechanisms are implicated in its deregulation in BC, including gene amplification and traslocations. *MYC* amplification plays a role in BC progression because it has been detected in the more aggressive phenotype of ductal carcinoma in situ [26] or in invasive processes [27–29].

Gene amplification of *CCND1* has been observed in a subgroup of BCs with poor prognosis and associated with resistance to tamoxifen [30]. Region of amplification is 11q13, and *CCND1* acts as a cell cycle regulator, promoting progression through the G_1_-S phase [31].

Higher *ESR1* gene amplification is found in BC with *CCND1* gene amplification in comparison with tumours without *CCND1* gene amplification [32]. Amplification of *ESR1* has been associated with negative ER [32]. The gene *TSPAN1* (on 1p34.1) has been found deleted in metastasizing BC and might represent an important TSG [33]. Another gene, *EMSY* was found involved in sporadic BC. *EMSY* amplification has been shown to be associated with a poor prognosis [34].

Compared to non-metastatic invasive ductal carcinoma, metastatic invasive ductal carcinoma showed a unique pattern of CNAs, including gains at 2p24-13, 2q22-33, 9q21-31, 12q21-23, 17 q23-25 and loses at 11q23-ter, 14q23-31, 20p11-q12, 2q36-ter, 8q24-ter, 9q33-ter, 2p11-q11, and 12q13 [35, 36].

Table 1 reports a synthesis of the considered mutated genes in BC, with their genetic alterations due to CNs.

**Table 1:** Genes mutated and their alterations in BC

Genes	Genetic alterations	References
MYC	Amplifications and translocations	[25–29]
CCND1	Amplifications and Translocations	[30]
HER2	Amplifications	[15–18]
TOP2A	Amplifications or Deletions	[22, 23]
*PIK3CA, MET*	Amplifications	[24]
*PNRC1*	Deletions	[15]
*DBC1* and *DEC1*	Amplifications or Deletions	[15]
*TSPAN1*	Deletions	[33]
*EGFR*	Amplifications	[19, 20]
ESR1	Amplifications	[32]
*EMSY*	Amplifications	[34]

#### Experimental methods

Current experimental methods for the identification of CNA include cytogenetic techniques, microarrays, and sequencing-based computational approaches.

Karyotyping is a cytogenetic technique performing a standardized and effective single cell screening in order to identify significant genomic aberrations in pathological and in normal samples.

In a standard karyotyping, a dye like Giesma or Quinacrine is used to stain bands on the chromosomes. Each chromosome presents banding pattern for detecting CNAs. Thus, any alteration in banding pattern represents a CNA [37].

Spectral karyotyping (SKY technique) is a novel technique for chromosome analysis [37], based on the approach of the fluorescence *in situ* hybridization technique (FISH). Sky refers to the multicolour-FISH technique where each chromosome is represented with different colours (a dye with different fluorophores). This technique is used to identify CNAs in cancer cells and in other disease conditions when other techniques are not enough accurate [37].

Resolution is the main limitation of both techniques, the chromosome profile obtained by karyotyping being not enough sensitive to notice short and relevant abnormalities [38].

Hybridization-based microarray approaches, including array comparative genomic hybridization (array CGH) and Single Nucleotide Polymorphism (SNP) microarrays, have been used as an alternative technology to conventional cytogenetic approaches [39]. They are able to infer CNAs (amplifications and deletions) compared to a reference sample. Array CGH platforms compare quickly and efficiently two labelled samples (different fluorophores - test and reference). Denaturation of the DNA in single stranded allows the hybridization of the two samples to microarrays containing DNA sequence probes of known genome position (e.g. bacterial artificial chromosomes, cDNAs, or more recently, oligonucleotides). By using a fluorescence microscope and a dedicated computer software, the signal ratio of different coloured fluorescents is measured in order to identify chromosomal differences between the two sources. An important consideration is the consequence of the reference sample on the CN profile. A comprehensive-characterized reference is the key for the correct interpretation of array CGH data [40].

SNP-arrays have a higher resolution than CGH-arrays, and can be used to identify allele-specific information. SNP microarray has few key differences from CGH technologies. Probe designs are specific to single-nucleotide differences between DNA sequences.

Ultimately, next generation sequencing (NGS) have replaced microarrays as the platform for discovery and genotyping, and present considerable computational and bioinformatics challenges.

#### Computational methods

We can summarize CNA analysis from microarray in three steps: 1) normalization, 2) probe-level modelling, and 3) CN estimation [41].

The target of normalization is to remove non relevant effects, such as the GC content of the fragment amplified by PCR, technical variations between arrays occurring from differences in sample preparation or labelling, and array production or scanning differences [42].

Probe-level modelling is usually performed at two levels: single locus and multilocus. Single locus modelling measures the CN of a specific target fragment or DNA probe locus in order to produce a raw fragment CN. Multilocus modelling combines the raw CNs of neighbouring fragments or DNA probe loci into a “meta-probe set” which determines the CN of the whole region [41, 42].

Computerized methods to estimate CNs (e.g. segmentation) performs the detection of break points which separate neighbouring regions based on the Log ratio of probe intensity [41, 42].

Several methods are suitable for analysing CNA on microarray data.

i) The first CNA analysis method has been developed by Affymetrix: Chromosome Copy Number Analysis Tool [43]. Normalization is performed by quantile normalization. Modelling uses robust multichip average. CN estimation can be done subsequently with an arbitrary algorithm.
ii)  DNA-Chip Analyzer (dChip) [44] normalizes using an invariant set method which corresponds to a normalization of the arrays based on the identification of a common baseline array and on adjustment of all the other arrays relative to the baseline array. Modelling is based on a model-based expression index (MBEI) for single-locus. This output is then used by a Hidden Markov Model (HMM) to infer CNs [44].
iii)   Copy Number Analyser for GeneChip arrays (CNAG) [45] normalizes the arrays in order to have the same mean signal intensity for all autosomal probes. This allows fragment probes comparable between arrays to be obtained. The signal intensity ratios is corrected for the differences in PCR product length and GC content. An HMM algorithm is applied to infer CNs along each chromosome.
iv)   Birdsuite's Birdseye [46] normalizes using quantile normalization. Modelling and segmentation are performed together at the multi-loci level. HMM estimates CNs.
v) Copy-number estimation using Robust Multichip Analysis (CRMA) [47] has been developed as an extension of the RMA model. Normalization is obtained by allelic cross-hybridization correction (ACC). Modelling uses robust multichip average (RMA). CNA analysis can be done using an arbitrary segmentation algorithm.

Given the different existing computational methods for CNA detection using SNP arrays, researchers have the problem to choose the optimal tool for their analyses.

With the aim of offering a support to bioinformatics researches and to answer to their emerging needs to choose among different CNA detection algorithms, the CNV Workshop was developed [48]. It represents the first cohesive and convenient platform for detection, annotation, and assessment of the biological and clinical significance of structural variants [48]. The purpose of the platform is to process data from a wide variety of SNP arrays, and to implement different normalization and CN estimation algorithms.

Since one of the main problem in the choice of the tool is the detection of discrepancies among different platforms [49], some studies have compared the different analysis using the same data set. Although limited to few methods, due to the high computational cost, several studies allowed the assessment of advantages and disadvantages of some techniques [49–51].

Baross et al.[49] found that CNAG, dChip, CNAT and GLAD are suitable for high-throughput processing of Affymetrix 100 K SNP array data for CN analysis. However, the tools revealed considerable variations in the numbers of putative CNA. dChip found more CNA than the other tested tools. The highest rate of false positive candidate deletion calls was produced by CNAG. In general, the performance of all tools in the detection of single copy deletions was better than that of single copy duplications. The authors recommend also the use of reference data set for accurate analysis, processed in the same laboratory and ideally from samples with an ethnic composition similar to the sample set.

Eckel-Passow et al. [50] provided a description of four freely-available software packages (PennCNV, Aroma. Affymetrix, Affymetrix Power Tools (APT), and Corrected Robust Linear Model with Maximum Likelihood Distance (CRLMM)) that are commonly used for CNA analysis of data generated from Affymetrix Genome-Wide Human SNP Array 6.0 platform. APT obtained the best performance with respect to bias. However, PennCNV and Aroma.Affymetrix had the smallest variability associated with the median locus-level CN.

Zhang et al. [51] assessed four software programs currently used for CNA detection: Birdsuite (version 1.5.2), PennCNV-Affy (a trial version), HelixTree (Version 6.4.2), and Partek (Version, 6.09.0129). They evaluated the accuracy in detecting both rare and common CNVs in the Affymetrix 6.0 platform. They found considerable variations among the programs in the number of CNAs. Birdsuite obtained the highest percentages of known HapMap CNAs containing more than twenty markers in two reference CNA datasets. In the tested rare CNA data, Birdsuite and Partek had higher positive predictive values than the other tools.

Other methods exist for analysing CNA on NGS and they are not described in this review. However, most of the more recent algorithms for CNA discovery are modelled on computational methods which were first used to analyse capillary sequencing reads and fully sequenced large-insert clones [39].

#### Therapeutic approach

A future challenging direction is the discovery of gene CN changes for the development of therapies. For example, duplication of one gene encoding a specific receptor can be associated with a particular pathology. Thus, compounds that down regulate receptor expression may lead benefit in patients.

Cancer is the prime case in which CNAs have been shown to drive disease [52] and therapies where overexpressed or amplified oncogenic drivers are targeted have been already considered. In particular, in BC, the gene encoding epidermal growth factor receptor (*EGFR*) results to be amplified, and small molecules such as gefitinib, erlotinib, lapatinib, and cetuximab have been applied to inhibit *EGFR* with benefits for patients [53, 54].

*ERBB2,* encoding HER2, is amplified in 30 % of BC [17, 55]. In the therapy of HER2-amplified BC, trastuzumab, an anti-HER2 antibody, has been used [56]. Pertuzumab, a humanized monoclonal antibody, binds HER2, and like trastuzumab, it stimulates antibody-dependent, and cell mediated-cytotoxicity [57]. Pertuzumab and trastuzumab binds to different HER2 epitopes acting in the same way. When given together, they operate reinforcing antitumor activity [58].

These proven benefits, although limited to few genes involved in BC, raise the exciting possibility that targeting amplified disease drivers may offer opportunities for therapy development in BC where effective treatments are still limited.

## Epigenetic alterations in BC

### DNA methylation and histone modifications

DNA methylation and histone modifications play a crucial role in the maintenance of cellular functions and identity. In particular, the main cellular networks affected by epigenetics are cell cycle, apoptosis, DNA repair, detoxification, inflammation, cell adhesion and invasion.

In cancer, the DNA methylation and histone modifications are perturbed, leading to significant changes in GE, which confer to the tumoral cells advantages in proliferation and maintenance of tumoral phenotype. For instance, the genomic inactivation of a tumor suppressor gene (p53, BRCA1,…) or the activation of an oncogene (i.e., Myc) contribute to the malignant transformation. Epigenetic changes differ from genetic changes mainly because they occur at a higher frequency than genetic changes, they are reversible upon treatment with pharmacological agents and occur at defined regions in a gene.

DNA methylation refers to the addition of a methyl group (−CH_3_) covalently to the base cytosine (C) in the dinucleotide 5′-CpG-3′. CpGs islands are in the promoter region of many genes [59, 60]. Most CpG dinucleotides in the human genome are methylated, and often leads to silencing of GE. The observation that CpGs islands of housekeeping genes are mainly unmethylated, and the methylation is associated with loss of GE led to the hypothesis that DNA methylation plays an important role in regulating GE [59, 60].

**Fig. 2:**
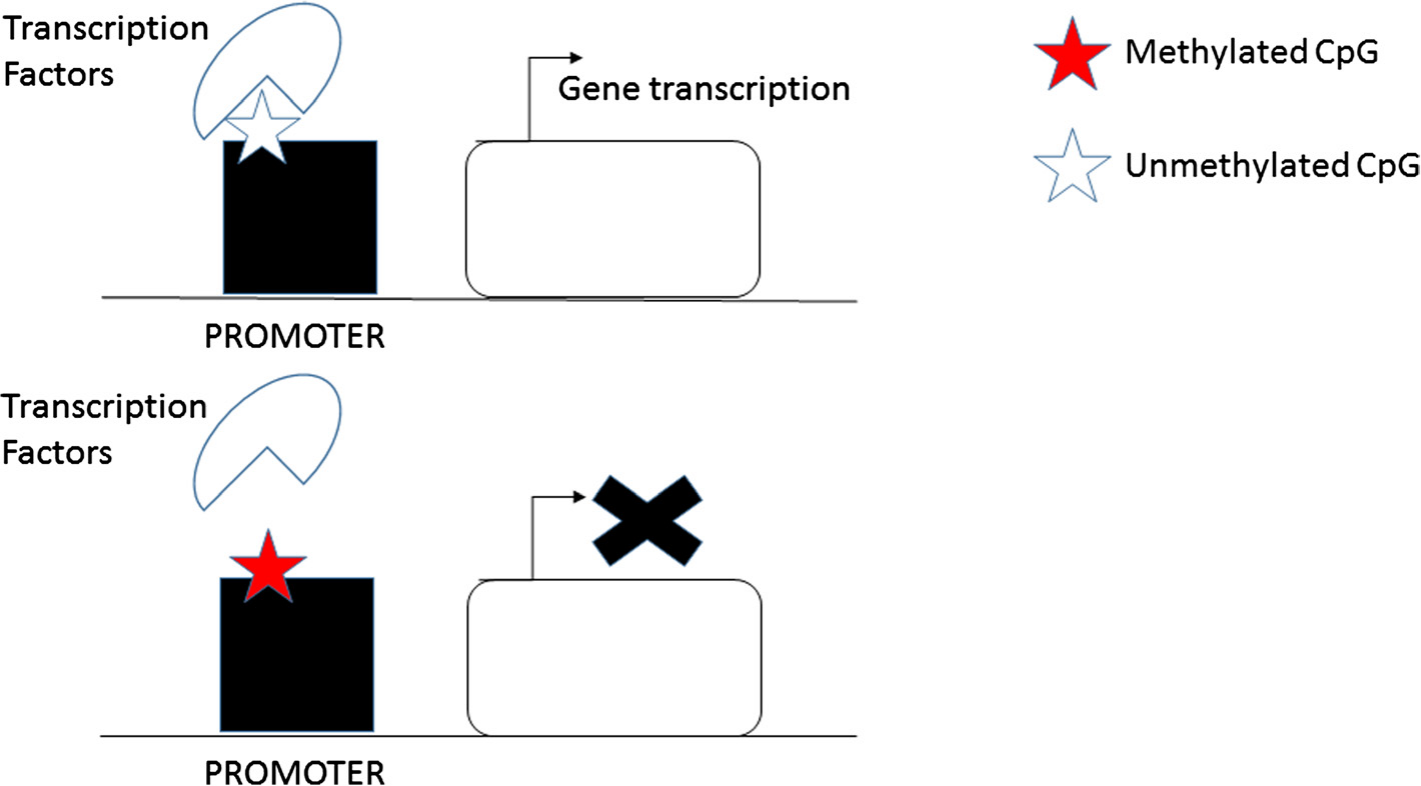
DNA methylation regulating GE. Methylated CpG restricts the binding between transcription factor and the gene promoter. Unmethylated CpG allows accessing of transcription factors to the gene promoter

Figure 2 shows how DNA methylation affects GE. Methyl groups in the recognition elements of transcription factors inhibits the binding of transcription factors to DNA, thus resulting in reduced transcriptional activity.

Histones are considered DNA-packaging protein components of chromatin, able to regulate chromatin dynamics. In fact they are subjected to several post-translational modifications, occurring at the amino-terminal end of the histone tail protruding from the surface of the nucleosome [61]. The modifications of histone tails, including lysine acetylation, lysine and arginine methylation, lysine ubiquitylation, phosphorylation, sumoylation, and ribosylation, can significantly affect the expression of genes in a dynamic manner [61]. The most studied histone epigenetic alterations are acetylation/deacetylation, and methylation/demethylation. In BC, abnormal histone modification and DNA hypermethylation are frequently associated to epigenetic silencing of tumor suppressor genes and genomic instability [62, 63].

#### Biological insights

The distribution of methylated and unmethylated CpGs in the genome shows different patterns of methylation confirming tissue-specific manner [64].

DNA methylation biomarkers for early detection and prognosis of cancer have been studied in the last years. Table 2 shows genes differentially methylated in BC.

**Table 2:** Genes differentially methylated in BC

Genes	Biological effects	References
*RASSF1A* and *CCND2*	Significantly more methylated in the ER+ than ER− cancers	[69]
*PGR, TFF1, CDH13, TIMP3, HSD17B4, ESR1* and *BCL2*	The inverse correlations were found between their hypermethylation and ER expression	[70]
*ESR1, TGFBR2, PTGS2* and *CDH13*	They were associated with PR expression	[70]
*FAM124B*, *ST6GALNAC1*, *NAV1* and *PER1*	The methylation status were quite different between ER+/PR+ and ER−/PR− BC	[71]
*RASSF1A, CCND2, TWIST, HIN1*	Low levels of methylation were detected in normal control samples	[74]
*CCND2, RASSF1A, APC* and *HIN1*	Able to distinguish between invasive carcinomas, fibroadenomas, and normal tissue	[66]
*ITIH5*, *DKK3*, and *RASSF1A*	Early detection of BC	[74]
*APC*, *BIN1*, *BMP6*, *BRCA1*, *CST6*, *ESR-b*, *GSTP1*, *P16*, *P21* and *TIMP3*	Able to distinguish between cancerous and normal tissues	[67]
*CST6*	Differentially methylated between BC and control plasma samples	[75]

Fackler et al. [65] found that promoter methylation of 4 genes *(*RASSF1A, CCND2, TWIST, HIN1*)* was more frequently detected in tumor than in normal tissue. In another study [66], 4 genes *CCND2, RASSF1A, APC* and *HIN1* were able to classify between invasive carcinomas, fibroadenomas, and normal tissue. 10 hypermethylated genes, *APC*, *BIN1*, *BMP6*, *BRCA1*, *CST6*, *ESR-b*, *GSTP1*, *P16*, *P21* and *TIMP3,* were identified to distinguish between cancerous and normal tissues [67].

Several studies provide strong evidence of DNA methylation signatures with prognostic role. DNA methylation status of the *PITX2* in BC cell lines is negatively associated with PITX2 mRNA expression and with poor prognosis [68].

Previous studies observed several candidate methylation sites that are associated with the hormone receptor status of BC. *RASSF1A* and *CCND2* were significantly more methylated in the ER+ than ER− BC [69], whereas the inverse correlations were identified between hypermethylation of the *PGR, TFF1, CDH13, TIMP3, HSD17B4, ESR1* and *BCL2* genes and ER status [70]. Hypermethylation of the *ESR1, TGFBR2, PTGS2* and *CDH13* genes was associated with PR status [70].

Li et al. [71] used 27 K arrays in a small sample of ER/PR+ and ER/PR BC samples, and identified and validated four genes: *FAM124B* and *ST6GALNAC1* were significantly hypermethylated, and *NAV1* and *PER1* were significantly hypomethylated in ER+/PR+ BC.

Fang et al. [72] used genome wide analysis to characterize BCs based on their metastatic potential. The study found a coordinated methylation of a large number of genes discovering a “methylator” phenotype. The methylator phenotype was associated with low metastatic risk and survival.

Identification of promoter methylation of biomarker genes in the DNA of bodily fluids, like serum or plasma, is a rapidly growing research field in cancer detection.

The principle is based on evidence that solid malignant tumors release significant amounts of cell-free DNA into the bloodstream through cellular necrosis or apoptosis [73].

The analysis of the methylation patterns of cell-free DNA by a blood-based test could become a screening tool. In particular, DNA methylation in circulating free DNA from blood of BC was investigated. ITIH5*,* DKK3, and RASSF1A promoter methylation from serum were identified as candidate biomarkers for the early detection of BC [74].

*CST6* has been identified by two independent dataset as being differentially methylated between BC and control plasma samples [75].

*SOX17* promoter is highly methylated in primary BCs, in circulating tumor cells isolated from patients with BC, and in corresponding cell-free DNA samples [76].

Similar studies on plasma identified hypermethylation status of KIF1A [77], and HYLA2 locus [78] in BC suggesting methylation level in blood having a power to distinguish very early BC cases from controls.

Non-invasive technique such as blood-test screening is a more suitable and cost-efficient methodology compared to mammography and magnetic resonance imaging.

Actually, in clinical use no specific methylation biomarker has been yet validated, due to the reduced number of matched normal DNA samples in cohorts.

Characterizing more than 880 human BC, Elsheikh et al. have demonstrated that histone acetylation and methylation patterns represent an early sign of BC [79]. Low levels of acethylated lysine and methylated lysine and arginine were described to have prognostic value, i.e. of triple-negative carcinomas and HER2-positive BC subtype [79, 80].

#### Experimental methods

In recent years three major technologies have been employed in DNA methylation analysis: chemical treatment with bisulphite (BS), methylation-specific enzyme digestion, and affinity enrichment [81, 82].

The first category includes an assay to characterize methyl cytosine by treatment of genomic DNA with BS. BS treatment converts unmethylated cytosine residues to uracil, without recognizing methyl cytosine residues, which are protected against this treatment.

Methylated and unmethylated DNA can be distinguished by the employment of sequence analysis (e.g. NGS, microarray). PCR amplicons created after BS conversion can be hybridized to microarrays containing methylation-specific oligonucleotides (MSO; 19–23 nucleotides) to query DNA methylation status [83]. BS-based methods cannot distinguish between methylcitosine and other variants (e.g. hydroxymethylcytosine) [84].

The second category includes methylation-sensitive restriction endonucleases, which distinguish sequences based on methylation status; furthermore methylcytosine could be identified by immunoprecipitation with antibodies or by affinity purification on methyl-binding protein beads.

Restriction endonucleases and microarray are also combined for high-throughput examination of the methylation status [85, 86]. A limitation in utilizing restriction endonucleases is that enzymes identify only a limited fraction of genome CpG sites [81, 82]. A methodology [87] with multiple enzyme-mediated restrictions was proposed, leads to a better coverage of all CpG dinucleotides in mammalian genomes.

A third category, enrichment techniques, include methylated DNA immunoprecipitation (MeDIP). Genomic DNA is immunoprecipitated with a monoclonal antibody that specifically identifies 5-methylcytidine. The immunoprecipitated fraction can be detected by PCR in order to identify the methylation state of individual regions [88].

A combination approach of MeDIP and methylation-sensitive restriction endonucleases was developed, promising to quickly compare methylomes at lower cost [89]. Alternatively, MeDIP can be combined with large-scale analysis (e.g. microarrays) [88].

Many of the techniques proposed for DNA methylation profiling can be combined with NGS technologies [90].

#### Computational methods

Bioinformatics research has been focused on the prediction of DNA methylation information with a dual purpose: i) accurate DNA methylation predictions could replace experimental data, and ii) DNA methylation prediction algorithms from training data can give additional information of an epigenetic mechanism.

A large number of computational predictive models have been developed to identify CpG dinucleotides methylated or unmethylated [91, 92], CpG islands (or CpG-rich regions) methylated or unmethylated [93, 94], and CpG islands (or CpG-rich regions) differentially methylated in different tissue/cell types or phenotypes [95]. Most of them use DNA sequence characteristics combined with a machine-learning algorithm.

Combination approaches of computational and experimental methods can speed up genome-wide DNA methylation profiling and detect crucial factors or pathways driving DNA methylation patterns. However DNA methylation prediction shows some difficulties: i) DNA methylation of the sampled cells need to be averages across cells, ii) there are differences across tissues, iii) DNA methylation can have unstable position, and iv) can be not well located in a genomic locus [96, 97].

A key step for accurate computational predictive models is a correct features selection.

The features can be grouped into two categories: genetic and epigenetic features. Given a region of interest (a CpG island or a genomic region around a particular CpG dinucleotide), the genetic features include: i) general features of the region of interest (e.g., length, and distribution of the CpG dinucleotides in the region), ii) DNA sequence composition of the region of interest, iii) patterns of conserved transcription factor binding sites or conserved elements within or near the region of interest, iv) structural and physicochemical properties of the region of interest, v) functional annotations of nearby genes, vi) single nucleotide polymorphisms of the region of interest, and vii) the conservation of the region of interest among species [98].

Epigenetic features are also crucial in order to fully characterize DNA methylation status.

DNA methylation, as an epigenetic phenomenon, is affected by some other epigenetic factors, such as histone methylation and histone acetylation.

Statistical methods related to differential DNA methylation data analysis cover a number of different approaches. In particular, these methods are accessible to the user by Bioconductor/R. Table 3 shows some methods and packages currently available for methylation differential analysis, such as Wilcoxon rank sum test (implemented in methyAnalysis package) [99], *t*-test (implemented in methyAnalysis, CpGAssoc, RnBeads, and IMA package [99–102]), Kolmogorov-Smirnov Tests ([100]), permutation test (implemented in CpGAssoc package [101]), empirical Bayes method (implemented in RnBeads, IMA and minfi package [101–103]), and bump hunting method (implemented in bumphunter and minfi package [104, 105]).

**Table 3:** Packages and methods for methylation differential analysis

Package	Method	References
methyAnalysis	Wilcoxon rank sum test	[100]
methyAnalysis, CpGAssoc, RnBeads, and IMA	*t*-test	[100–102]
-	Kolmogorov-Smirnov test	[104]
CpGAssoc	permutation test	[101]
RnBeads, IMA and minfi	empirical Bayes	[102, 103, 105]
bumphunter and minfi	bump hunting	[105, 106]

Wilcoxon rank sum test detect statistically significant sites according to the absolute difference between the average methylation levels of the analysed groups [106, 107]. This method can have a limitation in case of low or unbalance number of samples groups [107].

*t*-test is statistically inefficient in the presence of heterogeneity of methylation variability and shows many false positives, particularly for studies with smaller sample sizes [108, 109].

Kolmogorov-Smirnov test is another commonly used test that quantifies distributional differences. However, the Kolmogorov-Smirnov test considers each CpG marker as a sampling unit and its naive application is not valid [110, 111].

Permutation test is a resampling-based nonparametric test which permutes data following the null hypothesis of equal data distributions between groups [112].

Different number of empirical Bayes models were proposed for differential methylation analysis, with different statistical distribution assumptions [113]. Teng et al. [113] constructed five empirical Bayes models based on either a gamma distribution or a log-normal distribution, for the detection of differential methylated loci. They observed that log-normal, rather than gamma, could be a more accurate and precise method.

Bump hunting method used in bumphunter and minfi packages based the correlations of methylation levels between nearby CpG locus, and, for each locus, a linear model was used to estimate the coefficient of difference in methylation levels between the cancer group and the normal groups [105, 107].

A comparison study among these six statistical approaches was proposed [114]. Finally, different approaches were recommended for different applications: the bump hunting method is better for small sample size; the empirical Bayes methods are suggested when DNA methylation levels are independent across CpG loci, while only the bump hunting method is suggested when DNA methylation levels are correlated across CpG loci. All methods are found suitable for medium or large sample sizes [114].

#### Therapeutic approach

Cancer was the first group of diseases to be associated with DNA methylation. Numerous genes have been identified as being differentially methylated in BC, with a crucial roles in DNA repair, apoptosis, hormone receptor, and cell cycle. These TSGs may be good therapeutic targets through regulation of methylation activity by DNA methyltransferase inhibitors. Human DNA methylation is catalysed by enzymes of the DNA cytosine methyltransferases family including DNMT1, DNMT3A, DNMT3B and DNMT3L [115]. A lower DNA methyltransferase activity increases expression of silenced genes such as TSGs reactivating expression of key genes.

Previous studies [116, 117] were reported in BC focusing on action of DNA methyltransferase compound inhibitors.

Key targets for potential DNA demethylation agents are DNA methyltransferase inhibitor 5-aza-2′-deoxycytidine (decitabine), zebularine, and SGI-110 [117]. The mechanism of action of these pro-drugs is similar since they need to be incorporated into DNA to act as inhibitors of DNMTs [115].

Decitabine shows activity against hematologic malignancy and low-dose correlates with changes in GE induced by a reduction in DNA methylation.

A phase I clinical and pharmacodynamic trial was proposed in order to assess the feasibility of delivering a dose of decitabine combined with carboplatin [116]. Decitabine showed some limitations for treatment of advanced solid tumors (e.g. BC): i) weak stability, ii) lack of specificity for cancer cells, and iii) rapid inactivation by the action of cytidine deaminase [117].

Zebularine and SGI-110 are more selective for cancer cells and have higher resistance to deamination. In particular, Zebularine [118] showed an antitumor effect in a mouse model. In zebularine-treated mices, the oral treatment with zebularine showed a significant delay in tumor growth [118]. In combination with decitabine, zebularine has proven a significant inhibitory effect on cell proliferation and colony formation in MDA-MB-231 BC cell line through induction of ER alpha and PR mRNA expression [119]. Unfortunately, toxicity remain its main limitation [118].

SGI-110 [120], a 5′-AzapG-3′ dinucleotide, induces expression of the *p16* tumor suppressor gene, and inhibit tumor cell growth. This short oligonucleotide is resistant to cytidine deaminase deamination which may potentially increase its resistance, enhance bioavailability, and make the drug more efficacious.

DNA methyltransferase inhibitors can have side effects as the concomitant activation of both TSGs and OGs. The combination of chemotherapeutic agents and of DNA methyltransferase inhibitors could be efficacious [115].

Although the benefits of DNA methyltransferase inhibitors were demonstrated, toxicity, lack of specificity and low stability are issues to be solved in order to improve BC treatment [121].

Histone acetylation process is controlled by the balanced activity of histone acetyltransferases and histone deacetylases (HDACs). The HDAC family is divided into zinc-dependent enzymes (classes I, IIa, IIb, and IV, of which there are 11 subtype enzymes) and zinc-independent enzymes (class III, also called sirtuins), requiring NAD^+^ for their catalytic activity. Over the past decade, a number of HDAC inhibitors have been designed and synthesized, based on HDAC chemical structures. Some of these HDAC inhibitors are able to modify the chromatin structure, causing re-expression of aberrantly silenced genes, which in turn is associated with growth inhibition and apoptosis in cancer cells [122]. In ER-negative BC, the treatment with specific HDAC inhibitors reactivates ERα and progesterone receptor (PR) gene expression, which are known to be aberrantly silenced in BC. Preclinical studies of HDAC inhibitors combined with DNMT inhibitors or with anti-tumoral treatment (i.e., tamoxifen) have demonstrated a higher safety, tolerability and clinical effectiveness than single treatment [123, 124].

### microRNA deregulation in BC

#### Biological insights

miRNAs are small noncoding RNAs (20–22 nucleotides long) that are excised from longer (60–110 nucleotides) RNA precursor [105, 106] and act in different biological functions including development, proliferation, differentiation and cell death [125, 126]. miRNAs are major regulators of GE. Many evidences indicate that their deregulation is associated to several steps of cancer initiation and progression. In comparison with other approaches targeting single genes, they are certainly more stable thanks to their small size [127], and are able to discriminate different BC subtypes.

Blenkiron et al. found deregulated miRNAs between basal and luminal BC [128]. Iorio et al. [129], Lowery et al. [130] and Mattie et al. [131] identified miRNAs that were able to classify ER, PR and HER2/neu receptor status, respectively. Gregory et al. [132] found *miR-200* associated with the BC luminal subtype. Reduced expression of *miR-145* and *miR-205* was found to play a role in basal like triple negative tumours (ER-/PR-/HER2-) while are normally expressed in normal myoepithelial cells [133].

miRNAs can be also prognostic and predictive biomarkers. Zhou et al. [134] found *mir-125b* as useful indicator for poorly response to taxol-based treatments in vivo. The overexpression of *miR-181a* has been correlated with lymph node metastasis [135]. *miR-106b-25* expression was proven significantly predictor of good relapse time [136], while *miR-375* was found negatively regulate ER expression [137].

miRNAs with a role in metastasis in BC include *miR-7* [138, 139], *miR-17/20* [140, 141], *miR-22* [142–144], *miR-30* [145, 146], *miR-31* [147–149], *miR-126* [150], *miR-145* [151], *miR-146* [152], *miR-193b* [153], *miR-205* [154], *miR-206* [155], *miR-335* [156], *miR-448* [157], *miR-661* [158] and *let-7* [159].

miRNAs can be easily extracted and detected from blood [160], circulating exomes [161], saliva [162, 163], and even sputum [164, 165]. Several studies demonstrated that circulating miRNAs reflect the pattern observed in the tumour tissues (e.g. [167]), thus opening the possibility to use circulating miRNAs as biomarkers for diagnosis and prognosis. Lodes et al. [166] provided an evidence on using serum miRNAs as biomarkers to discriminate between normal and patients in many cancer diseases including breast, prostate, colon, ovarian, and lung cancer. They showed that it is sufficient 1 mL of serum to detect miRNA expression patterns, without the need of amplification techniques. Recently, an analysis of circulating miRNAs have led to identify *mir-21*, *miR-92a* [167, 168], *miR-10b*, *miR-125b*, *miR-155*, *miR-191*, *miR-382* [169] and *miR-30a* [170] as candidate biomarkers for early detection of BC. Circulating miRNAs have also been associated with disease prognosis and response to treatment. Madhavan et al. [171] found circulating miRNAs as marker of disease free survival and overall survival. Plasma *miR-10b* and *miR-373* were found associated with the development of metastases [172] while *miR-125b* [173] and *miR-155* [174] have been found correlated to chemotherapy response.

Table 4 reports a synthesis of the considered miRNAs deregulated in BC, with their principal biological effects.

**Table 4:** miRNAs deregulated in BC

MiRNAs	Biological effect	References
*miR-200*	It associated with the luminal subtype	Gregory et al. [132]
*miR-145 and miR-205*	It associated with basal like triple negative tumours	Sempere et al. [133]
*miR-125b*	It can predict poor response to taxol-based treatment *in vivo*	Zhou et al. [134]
*miR-181a*	It correlated with lymph node metastasis	Taylor et al. [135]
*miR-106b-25*	It can significantly predict a good relapse time	Smith et al. [136]
*miR-31*	It controls metastasis and increases the survival of patients	Valastyan et al. [147]
*let-7*	It suppressed metastasis	Yu et al. [159]
*miR-375*	It negatively regulate ER expression	de Souza et al. [137]
*miR-10b, miR-155*	They correlate with metastasis	Mar-Aguilar et al. [169]
*miR-21*	It associated with cell migration and invasion	Si et al. [168]

#### Experimental methods

Many technologies for detecting miRNAs have been developed, including RT-PCR, *in situ* hybridization, microarray, and NGS [7].

RT-PCR is a sensitive and precise technology but it is also an expensive and low-throughput method [7].

*In situ* hybridization is based on labelled complementary strands for the sequences of interest (e.g. miRNA) in a portion or section of tissue [175]. The small size of the mature miRNA presents problems for conventional *in situ* hybridization methods and it is semi-quantitative.

Microarrays have several limitations as those due to background or cross-hybridization problems. Moreover, microarrays and other techniques can provide analyses only on known miRNAs [7].

Contrarily, sequence-based methods allow the identification of unknown miRNAs and early overcome other methods. Stark et al. [176], by using deep sequencing, discovered and quantified new miRNAs. Similarly, Farazi et al. [177], generated a miRNA signature able to differentiate ductal breast carcinoma *in situ*, invasive ductal breast carcinoma and normal tissue.

Also deep sequencing may be a powerful method to study circulating miRNAs. Several studies investigated correlations among miRNAs in the serum of BC with clinicopathological indices [178] and found miRNAs associations with overall survival [179].

Despite the high potential and promising results of these methods in clinical applications, there are still some problems that need to be addressed, e.g. the lack of inconsistency for some results between different studies. Standardization of procedures for sample conservation, preparation and/or processing [180], and the use of different quality controls for data normalization [181] could be effective in reducing these limitations.

#### Computational methods

There are two different approaches to examine both miRNA and mRNA expression profiles.

A first approach considers either a miRNA or an mRNA first, and then applies ad-hoc strategies, such as computational or experimental methods, in order to obtain miRNA-mRNA pair information [182, 183].

A second approach examines miRNA and mRNA regulatory pairs together [184–187].

Computational methods play important roles in the identification of new miRNAs. These methods can be divided into three major categories: 1) sequence or structure conservation-based, 2) machine learning-based method, 3) and non-comparative methods.

Sequence or structure conservation-based methods are based on sequence/structure conservation as techniques to find miRNAs. The principle is the nature of conservation across different species for most of the known miRNAs. Comparative genomics filter out sequence/structure conservation that are not evolutionarily conserved in related species [188]. Examples of such computational methods, focusing on the secondary structure of RNA and looking for conserved hairpin structures between related species, are Srnaloop [189], MiRscan [190], and miRseeker [191]. One of the first study related to these methods was by Lee and Ambros [192]. The authors, using bioinformatics techniques, searched for sequences conserved between the *C. elegans* and *C. briggsae* genomes. They focused on premiRNA sequences and secondary structures with similar characteristics to *lin-4* and *let-7*, the first two miRNAs found on that time.

Several web based software tools have been developed to find new miRNA genes, based on sequence and secondary structure similarities with known miRNAs [189–191, 193]. However, the limit of these approaches was demonstrated by Bentwich et al., showing the possibility that large quantity of nonconserved miRNAs could be missed by the use of this tool [194].

Free energy (or Gibbs free energy) can be used as feature for miRNA target prediction. It shows how strong the binding of a miRNA with its target is by predicting how the miRNA and its candidate target will hybridize. The free energy of miRNA-mRNA binding is normally assigned by the RNAfold program-Vienna RNA Package [195].

Machine learning-based methods do not necessarily depend on sequence conservation. A classifier is constructed on a training dataset, that contains a set of known miRNA sequences (positive training dataset), and on a set consisting of mRNAs, tRNAs and rRNAs (negative training dataset). The information given to the classifier can be, for instance, the position of the mature sequence or the folding energy. The classifier, by describing a candidate miRNA with this set of features, is able to predict true and false miRNA sequences [196]. The limit of this approach is the choice of negative set. As example, we do not know a priori if a particular sequence can generate functional miRNAs [197]. Several studies have tried to overcome this kind of problems with the use of only positive models [198, 199]. However, the results were poorer than those found by approaches that consider both positive and negative training sets [199].

Different classification methods are currently available based on machine learning, e.g. SVM, neural networks, HMM, and Naive Bayes (NB), and several tools based on machine learning have been developed and released to the research community, e.g. RNAmicro [200], MiRFinder [201], ProMir [202], MiRRim [203], SSCprofiler [204], HHMMiR [205] and BayesMiRNAFind [206].

Non-comparative methods use intrinsic structural features of miRNA, and include algorithms like PalGrade [207], Triplet-SVM [208], miPred [209], miR-abela [210], and HHMMiR [211]. These methods are able of detecting a large number of miRNAs that seem to be unique to primates.

Bentwich et al. [207] developed PalGrade by integrating bioinformatics predictions with microarray analysis and sequence-directed cloning. This approach allowed the detection of 89 human miRNAs, 53 of them being not conserved beyond primates.

Xue et al. [208] proposed an *ab initio* classification of real pre-miRNA from other hairpin sequences with similar stem-loop features. SVM was applied on these features to classify real vs pseudo pre-miRNAs achieving 90 % of accuracy.

Ng et al. [209] employed a Gaussian Radial Basis Function kernel (RBF) as a similarity measure for 29 global and intrinsic hairpin folding attributes. They tested the model on 123 human *pre-miRs* and 246 pseudo hairpins, reporting 84.55 %, 97.97 %, and 93.50 % in sensitivity, specificity and accuracy, respectively.

Sewer et al. [210] developed miR-abela to detect human miRNAs. They focused on particular properties of some genomic regions around already known miRNAs, and were able to predict between 50–100 novel pre-miRNAs, 30 % of them already found as new in other studies.

#### Therapeutic approach

miRNAs may have a crucial role in guiding treatment decisions. miRNAs can be therapeutic agents in cancer for two major characteristics: (1) their expression is deregulated in cancer compared to normal tissues, and (2) cancer phenotype can be changed by targeting miRNA expression [196].

Compared to gene profiles, miRNA-based therapeutics have several advantages, as for example their ability to target multiple genes, frequently in the context of a network. miRNAs regulating the network of genes and cellular pathways play a crucial role in BC pathogenesis and therapy.

There are two strategies for developing miRNA-based therapies: i) by the introduction of miRNA-mimic oligonucleotides, which mimic miRNA expression, up-regulating miRNA, and ii) by the introduction of miRNA inhibitor oligonucleotides to inhibit the expression of the miRNA of interest. However, some major obstacles for the use of miRNA therapeutics exist, including the tissue-specific delivery [211, 212], and the fact that erroneous targeting of miRNAs may cause toxic phenotypes [213].

For an effective drug-design of miRNA-targeted therapies in BC, it could be useful to understand the interplay between miRNAs and mRNAs leading to BC, thus studying the networks of gene controlled by each miRNA of interest. miRNA and their targets can form complex regulatory networks, and the comprehension of miRNA-target relation will help the development of personalized and tailored therapies [213].

### Gene expression deregulation in BC

#### Biological insights

GE profiling in BC has been widely demonstrated to generate different prognostic and diagnostic gene signatures. However, molecular tests have a potential not only for diagnosis but also for tailoring treatment plans, in particular with the aim of reducing resistance, non-response and toxicity [214]. Most of the tests either focus on gene expression microarrays or quantitative reverse transcription (qRT)-PCR analyses.

van't Veer et al. [215] obtained one of the prognostic signature for BC currently available on the market: MammaPrint. Microarray analysis of 78 BC patients with no systemic therapy led to the identification of a list of 70 genes able to predict the prognosis of the disease. The test was independently validated in a cohort of 295 early stage invasive BC, and results proved that the signature was an independent prognostic marker in BC [216]. A second independent validation study was performed by the TRANSBIG Consortium [217] in a cohort of 302 adjuvantly untreated patients, and was followed by additional validation studies [218–220]. MammaPrint was developed by Agendia, a laboratory in Amsterdam, approved in 2007 by the U.S. Food and Drug Administration (FDA) and then released commercially. This is a microarray-based test assessing the risk that a BC can metastasize to other parts of the body.

Paik et al. [221] developed Oncotype DX, a qRT PCR-based signature which measures the expression level of 21 genes (16 target + 5 reference genes). The test is able to predict chemotherapy benefits and the likelihood of distant BC recurrence. This is the first genomic biomarker assay which is commercially available for BC treatment as support of chemotherapy. Three separate studies containing 447 BC patients allowed to identify the 21-gene profile, which were divided into 16 target and 5 reference genes. The test was then validated using 668 node negative, ER positive, tamoxifen treated patients from NSABP B-14. An Oncotype DX Recurrence Score (ODRS) was defined and measured, as expression of a risk percentage for the development of distant metastases [222]. Oncotype DX was subsequently evaluated in the NSA BP-B20 trial, a study that explored the benefit of chemotherapy plus tamoxifen, and proved the accuracy of the biomarker. Currently, the Oncotype DX assay is performed in the licensed Genomic Health laboratory, which is the laboratory where the assay was developed.

Prediction Analysis of Microarray (PAM50), by using qRT-PCR assay, measures the expression of 55 (50 target and 5 reference genes) to identify the intrinsic subtypes of BC: luminal A, luminal B, HER2-enriched, and basal-like [223]. The gene signature was developed by analysing 189 BC samples, and was then validated on 761 BCs for prognosis and on 133 BCs for prediction of response to a taxane and anthracycline regimen [223]. NanoString’s Prosigna™ received a CE-mark designation for selling BC PAM50 in 2012, and received FDA clearance in 2013.

Genomic Grade Index (GGI) [4] is a 97 gene which measures the histological tumour grade. This test is based on the assumption that histological grade is a strong prognostic factor in ER positive BC. Sotiriou et al. [4] found that GGI gene signature is able to classify BC as histological grades I and III. They used 64 samples of ER-positive BC tumours to select genes that were differentially expressed (DE) between histologic grade I and III tumours, and to generate the gene signature. Data from 597 independent tumours were then used to evaluate GGI and to also demonstrate that GGI can separate histological grade 2 BC into low or high categories with different clinical outcomes. The results of the BIG-1-98 study (55 endocrine-treated patients) [224] demonstrated that the GGI is also a potential predictor of relapse for endocrine-treated BC patients. Ipsogen launched the MapQuant Dx (TM) genomic grade test by incorporating GGI. The test is currently used, in particular when tumor grade information can be decisive for prescribing a chemotherapy.

Immunohistochemical (IHC) assay Mammostrat [225] uses 5 immunohistochemical markers (SLC7A5, HTF9C, P53, NDRG1, and CEACAM5) to stratify patients on tamoxifen therapy into different risk groups, in order to inform treatment decisions. In the validation study, an analysis was performed on two independent data sets of 299 and 344 BC samples [226]. Clarient launched on the market the Insight® Dx Mammostrat® Breast Cancer Recurrence test in 2010.

Table 5 reports the considered commercially available tests, with their principal characteristics (e.g. number of genes, validation data sets).

**Table 5:** Current commercially available genetic test for BC and their principal characteristics

	Author	N. genes	Samples used to generate BC signature	Independent validation study	Laboratory
MammaPrint	van't Veer et al. [215]	70	− 78 BC patients	− 295 early stage invasive BC.	Agendia
				− 302 who had received loco-regional therapy but no systemic adjuvant therapy	
Oncotype DX	Paik et al. [221]	21	− 447 BC patients	− 668 node negative ER positive tamoxifen treated cases	Genomic Health
				− 651 BC samples: 227 had been randomly assigned to tamoxifen adjuvant therapy and 424 to tamoxifen plus chemotherapy	
PAM50	Parker et al. [223]	50	− 189 BC patients	− 761 patients (no systemic therapy), 133 (neoadjuvant chemotherapy)	NanoString’s Prosigna™
Genomic Grade Index (GGI)	Sotiriou et al. [4]	97	− 64 BC patients	− 597 BC	Ipsogen
				− 55 endocrine-treated patients.	
Mammostrat	Bartlett et al. [225]	5	− 466 BC patients	− 299 BC, 344 BC	Clarient

#### Experimental methods

Understanding GE and how it changes under normal and pathological conditions is necessary to provide information about the expressed genes. Large scale GE data provide the activity of thousands of genes at once.

Several techniques exist for studying and quantifying GE.

Traditional methods focus on measuring the expression of one gene at a time, as, for example, the Northern Blotting and the Real-Time Quantitative Reverse Transcription PCR (RT-PCR).

Northern blotting (called also RNA blot) was the first tool used to measure RNA levels, and, until the end of the 1990s, it was used extensively. It allows to quantify levels of mRNA by electrophoresis, which is able to separate RNA samples by size. The RNA of interest is revealed by a hybridization probe complementary to it. The first step of RNA blot is to denature the RNA into single strands. Hence, gel electrophoresis separates the RNA molecules according to their size. Subsequently, the RNA is transferred from the gel onto a blotting membrane, containing RNA bands originally on the gel. A probe complementary to the RNA of interest binds to a particular RNA sequence in the sample [227]. The RNA-probe complexes can thus be detected using a variety of different chemistries or radionuclide labelling.

RT-PCR is a major development of PCR technology, overcoming Northern blot as the method for RNA detection and quantification [228]. It enables to monitor and measure a targeted DNA molecule generated during each cycle of PCR process. In RT-PCR, the mRNA must be converted to a double-stranded molecule by using the enzyme reverse transcriptase. This phase is followed by quantitative PCR (qPCR) on the cDNA with the detection and quantification of amplified products [229]. The quantity of each specific target is obtained by measuring the increase in fluorescence signal from DNA-binding dyes or probes, during successive rounds of enzyme-mediated amplification. The limitation of this technique is the quantification of few genes at a time [229].

Several technologies such as microarray, Serial Analysis of Gene (SAGE), Cap Analysis of Gene Expression (CAGE) and Massively Parallel Signature Sequencing (MPSS) allow the mRNA expression data for hundreds of genes to be obtained in one single experiment [230].

The most commonly used technology to profile the expression of thousands of transcripts simultaneously is microarray. DNA microarray is an array of oligonucleotide probes bound to a chip surface [231, 232]. Labelled cDNA from a sample is hybridized to complementary probe sequences on the chip, and strongly associated complexes are identified by detection of fluorophore-, silver-, or chemiluminescence-labelled targets [231, 232].

Many variables influence the outcome of the experiments in microarray analysis, thus contributing to experimental errors and biological variations (for more details see [233]).

In contrast to microarray methods, sequence-based approaches directly determine the cDNA sequence [234]. SAGE [235], CAGE [236], and MPSS [237], all tag-based sequencing approaches, are based on Sanger sequencing technology.

The development of novel high-throughput DNA sequencing methods, such as RNA-Seq (RNA sequencing), has provided new approaches for both mapping and quantifying transcriptomes. It has clear advantage over existing approaches: RNA-Seq is not limited to the detection of transcripts that correspond to existing genomic sequence, and it is suitable to discovery genomic sequences that are still unknown [234].

In RNA-Seq analysis, RNA is converted to a library of cDNA fragments with adaptors attached to one or both ends. Each molecule is then sequenced in a high-throughput way in order to obtain short sequences (reads 30–400 bp). Following sequencing, the resulting reads are mapped to the genome in order to produce a genome-scale transcription map consisting of both the transcriptional structure and the level of expression for each gene [234]. Although RNA-Seq has many advantages with respect to the other methods, other issues must be overcome to achieve best practices in the measurement of gene expression, for instance, the lack of accurate methods able to identify and track the expression changes of rare RNA isoforms from all genes [234].

Table 6 reports a synthesis of the considered experimental methods for studying and quantifying GE, with their principal advantages and limitations.

**Table 6:** Principal experimental methods for GE quantification

Method	Pros	Cons
Northern Blotting	-Inexpensive	-low throughput
	-detecting transcript size	-semiquantitative
		-RNAase contamination
RT-PCR	-high sensitivity	-high variability
	-high sequence specific	-normalizaton methods
Microarray	-measurement of the activity of thousands of genes at once	-high cost
	-rapid	-analysis of Big data
	-don't require large-scale DNA sequencing	-high Background noise
Sanger sequencing technology	-low Background noise	-only a portion of the transcript
		-isoforms are generally indistinguishable from each other
		-Low throughput
RNA-seq	-measurement of the activity of thousands of genes at once	-High cost
	-require low amount of RNA	-Analysis of Big data
	-high reproducibility	
	-Low Background noise	

#### Computational methods

Microarray or RNA-sequencing technologies, as above reported, produce an overall design of all the transcriptional activity in a biological sample. However, these methods necessarily produce a large amount of data to be visualized, evaluated for their quality, normalized, filtered and interpreted.

Hence, the data originated by platforms (such as microarrays or RNA-seq) must be pre-processed. Pre-processing step is crucial to normalize the data and to clean biological signal values from experimental noise [238, 239].

Data must be also reduced prior to be used in advanced analysis, and this can be accomplished in two different ways: 1) by dimensionality reduction methods, that do not modify the original representation of data, and 2) by dimensionality reduction techniques which involve modification or loss of information from the original data. Among this second category, there are those methods based on projection (e.g. principal component analysis) or compression (e.g. using information theory) [240].

One of the most validated method of the first category is feature selection technique. It is often used to identify key genes able to separate the samples into different classes (e.g. cancerous and normal cells), and to remove irrelevant genes. Golub et al. [241] showed indeed that most genes are not significant in a problem of samples classification. However, feature selection is also important in order to obtain faster and efficient classification models, and to avoid over fitting.

Three categories of feature selection methods can be used: filters, wrappers, and embedded methods [242, 243].

Filter methods find subset of genes dependent on the class label, and do not consider the relevance of genes in combination with other genes [243]. Usually they are simple and fast.

Filter methods include correlation-based feature selection (CFS) [244], *t*-test [243, 245], information gain [243, 245], mutual information [246], entropy-based methods [247], Euclidian distance [245], signal to noise ratio [245], and significant analysis of microarrays [248].

Wrapper methods try to achieve the best combination of genes that may offer high classification accuracy. They include hybrid genetic algorithms [249], particle swarm optimization [250], successive feature selection (SFS) [251] and GA-KDE-Bayes [252]. However, this approach is less used, in particular in microarray analysis, due to its high computational costs [243].

Filter approach does not interact with the classifier, contrarily to wrapper and embedded techniques, usually resulting in lower performance.

An intermediate approach between the lowest results of the filter methods and the high computational cost of the wrapper methods is represented by the embedded method. With this method, the feature selection procedure is inbuilt to a classifier. Classification trees like ID3, random forest, and Support Vector Machine (SVM) based on Recursive Feature Elimination (RFE) are all examples of embedded methods [243, 245].

**Fig. 3:**
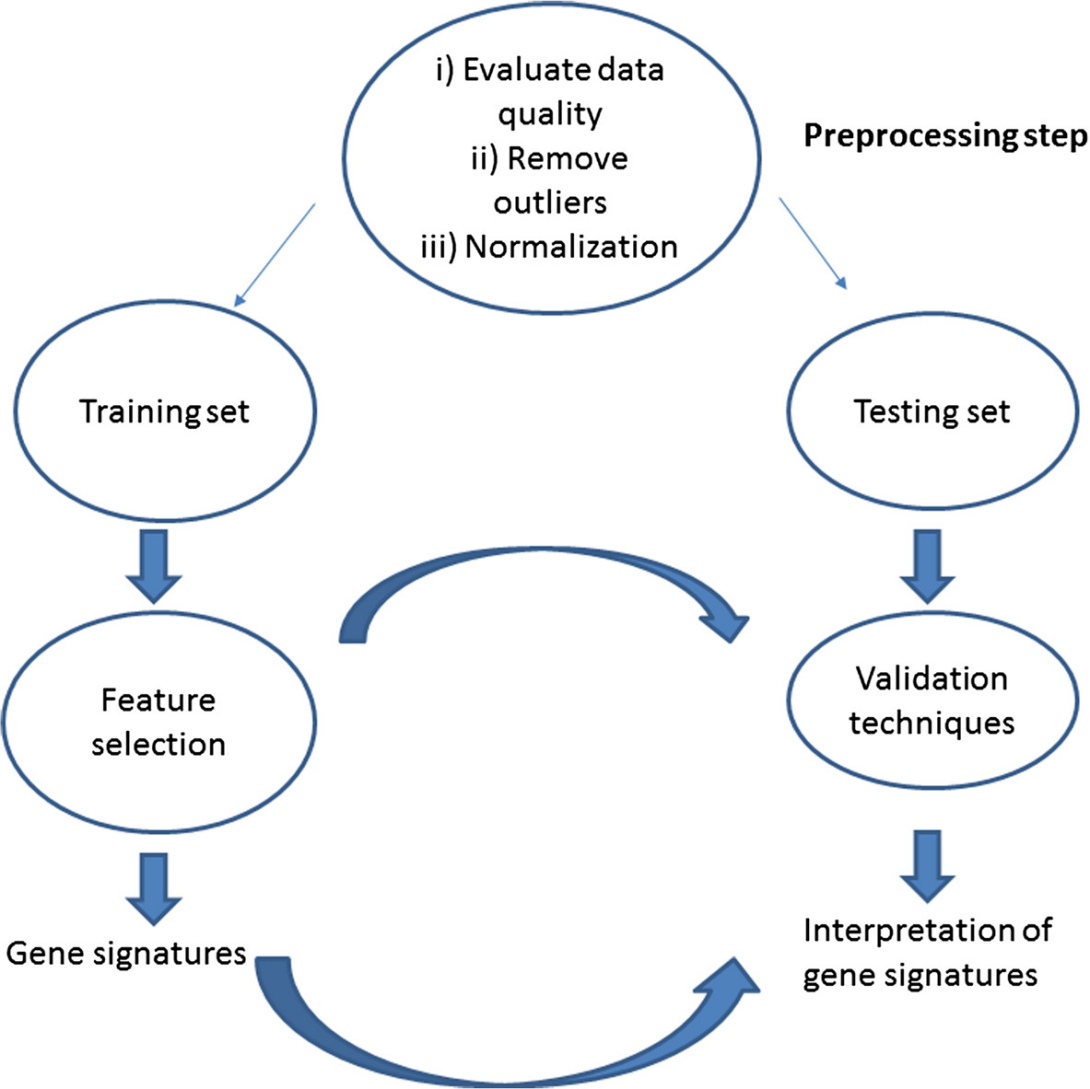
Feature selection model and validation. Feature selection acts on a training data set giving a gene signature. Gene signature is validated on a testing data set

The principle of the feature selection and validation techniques is shown in Fig. 3. A pre-processing step is performed: i) the quality of data is evaluated, ii) outliers are removed, and iii) data are normalized. Feature selection is performed. Usually, original data are divided into two data sets: a training set, subjected to the feature selection, and a testing set, used to evaluate the feature selection of the model with different validation techniques. Feature selection finds a subset of genes of interest, (e.g. a gene signature), and the validation of genes is performed. The most used validation techniques are cross-validation or leave one out validation [243], even if several studies suggested the use of a 10-fold cross validation because they give a more biased but less variable estimate than the leave-one-out error (e.g. [253]). When the feature selection of the model satisfies the required validation performance, the genes are defined and can be interpreted.

#### Therapeutic approach

Drug compounds that facilitate and control tightly therapeutic GE are a promising target. Transcriptional gene regulatory system has been encoded within several viral vectors (eg. Tetracycline-based systems can regulate GE of particular targets with the use of cell-type-specific promoters) [254].

The regulation of GE systems is an attractive target for gene therapy development, and potential applications have been assessed in a wide variety of preclinical laboratory models of disease. The first study was performed by Hallahan et al. [255], which described how TNF-a expression, under the control of the Egr-1 promoter, could be increased in response to ionizing X-ray radiation. This increase of TNF-a expression was associated with an improved control of tumour growth in comparison with X-ray radiation alone [255]. Advantages from induction of GE by ionizing radiation include reduction of damage to adjacent healthy tissues [254].

Kan et al. [256] have constructed a novel retroviral vector (MetXia-P450) encoding CYP2B6. This vector was used to transfect the human tumour cell lines HT29 and T47D. CYP2B6 metabolizes the prodrug cyclophosphamide (CPA) to produce phosphoramide mustard that cross-links DNA, thus leading to cell death. In order to evaluate safety and clinical response, MetXia-P450 entered Phase I clinical trials for nine BC patients and three melanoma patients with cutaneous tumours, with encouraging results.

Although viral vectors are very efficient for gene transfer, their uses are still limited by safety concerns [257]. As an alternative, non-viral BC gene therapy (e.g. naked DNA) is growing due to its safety profile, easy preparation procedures, and moderate costs. β-galactosidase (LacZ) expressing plasmid DNA has been successfully delivered in three patients by a needle-free jet injection to skin metastases from primary BC, and also to melanoma lesions in 14 patients. No side effects were observed. The transgene was detectable at messenger RNA (mRNA) and at protein levels in all patients.

### Copy number alterations and gene expression in BC

#### Biological insights

Several studies demonstrated that changes in DNA CN are translated into corresponding changes in GE [258, 259]. Although it is possible that changes in specific DNA sequences (i.e. centromeres or telomeres) can have directly negative consequences [260], the main responsible for the malignant phenotype has been proven to be the *gene dosage hypothesis*: alterations of gene copies change the expression levels of the involved gene [261].

**Fig. 4:**
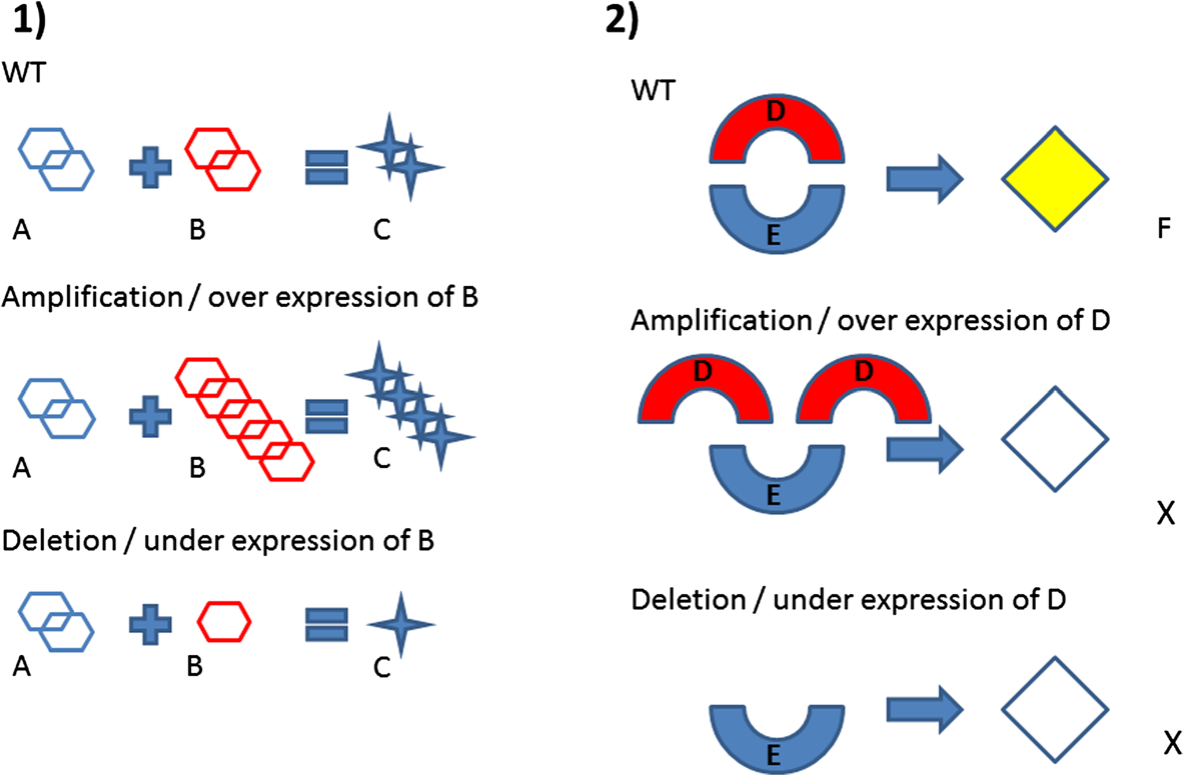
Consequences of gene dosage. **1**) WT cell: a correct number of gene copies and expression gives a correct production of C. Amplification/Over expression of B can increase the output. Under expression of B can diminish the production of C. **2**) WT cell: a correct number of gene copies and expression form a correct complex DE producing F. Amplification/Over-expression of D can interfere with stoichiometry of protein complex inhibiting F. Deletions/Under-expression of D not form complex DE and not produce F

Figure 4 shows the principle consequences of an altered gene dosage. Specifically, figure 4.1) shows: i) WT condition where a correct number and expression of A and B gives a correct production of C; ii) how the amplification/over expression of gene copies (e.g. B) can cause an increased dosage of a single gene (e.g. C), and iii) how a deletion/under expression of gene copies (e.g. B) can cause a decreased dosage of a single gene (e.g. C) [261]. Figure 4.2) shows how altered gene dosage can influence stoichiometry of protein complex DE that produces F. An amplification/over expression of protein D can inhibit the formation of protein complex DE, thus altering the pathway activity and the correct production of F. A deletion/under expression of protein D do not produce protein complex DE [262].

While useful information has been revealed by analysing GE profiles alone or CNA data alone, integrative analysis of CNA and GE data are necessary in order to have more information in gene characterization. Specifically, RNA data give information on genes that are up/down-regulated, but do not consider primary changes driving cancer from secondary modifications, such as proliferation and differentiation state. On the other hand, DNA data give information on amplifications and deletions that are drivers of cancer. Therefore, integrating DNA and RNA data can clarify genetic regulatory relationships in cancer cells [262]. It is interesting that transcriptional changes for 10–63 % of genes occur in amplified regions, and, for 14–62 % of genes, in regions of loss [263].

Several studies showed that gains (or losses) in DNA genomics have consequences in the expression levels of genes in the implicated regions, which are increases or decreased, respectively [264–266]. If we consider individual genes, the situation is more complicated. For instances, 14 % of down-regulated genes can appear within regions of DNA gain, while 9 % of up-regulated genes can occur in regions of DNA loss [266]. These findings suggest to take a particular attention in the integration of CNA and GE.

The Cancer Genome Atlas project [267] is generating multidimensional platforms including gene expression and CNA data for the same set of patients [263]. Although it is possible to perform analysis with unpaired data [263, 268, 269], the analysis is much more accurate when both types of data are available from the same patient. In this condition, the paired data analysis allows better statistical power and a reduction of false positives [270, 271].

Some studies have shown that integrating CNA information with GE data can often provide a powerful tool for identifying functionally relevant genes in cancer [e.g. 275–282]. Chen et al. [272] found a list of eighteen genes for which a strong correlation between CNA and GE exists, using signal-to-noise ratio (SNR). They found one particular gene, *RUNX3*, which is involved in the control of the *in vitro* invasive potential of MDA-MB-231.

Zhang et al. [273] identified an 81-gene prognostic CN signature that was found highly correlated with GE levels (Cox regression P < 0.05). This signature identified a subgroup of patients with increased probability of distant metastasis in an independent validation set of 113 patients.

Andre et al. [274] reported the level of mRNA expression, significantly correlated to the CAN, for *VEGF*, *EGFR*, and *PTEN*, using Algorithm Array CGH Expression integration tool (ACE-it). These genes could be targeted in triple-negative BC in clinical trials, and one of them, *E2F3*, can have a major role in a subset of triple-negative BC.

Hyman et al. [275] studied CNAs in 14 BC cell lines, and identified 270 differently expressed genes using signal-to-noise statistics (α value <0.05). 91 of the 270 genes represented hypothetical proteins or genes with no functional annotation, whereas 179 genes had available functional information.

Orsetti et al. [276] presented a study on CNA on chromosome 1, the prevalent target of genetic anomalies in BC, and the CNA consequences at the RNA expression level in BC. They identified 30 genes showing significant over-expression. A discriminating score was applied by comparing the expression levels of the subgroup of samples presenting amplification and the expression levels of the subgroup of samples without amplification.

Chin et al. [277] associated CNA and GE profiles of genes linked to poor treatment response. They identified 66 genes in these regions whose expression levels were correlated with CN, using Pearson's correlation (FDR < 0.01, Wilcoxon rank-sum test). Gene Ontology analyses of these genes showed that they are involved in nucleic acid metabolism, protein modification, signalling, and in the cell cycle and/or protein transport.

Chin SF et al. [278] evaluated genome-wide correlations between GE and CN by following an approach based on the Wilcoxon test. They showed strong statistical associations between either CN gain and over-expression (196 genes) or CN loss and under-expression (63 genes). Many well-known and potentially novel oncogenes and tumour suppressors were included in their analysis.

Table 7 reports a synthesis of the considered genes based on the integration of CNA and GE.

**Table 7:** Gene signatures obtained by the Integration of CNA and GE

Number of genes (gene signatures)	References
1	Chen et al. [272]
81	Zhang et al. [273]
4	Andre et al. [274]
270	Hyman et al. [275]
30	Orsetti et al. [276]
66	Chin et al. [277]
259	Chin SF et al. [278]

#### Computational methods

No experimental methods actually exist giving, in one single analysis, results about the integration of CNA and GE.

**Fig. 5:**
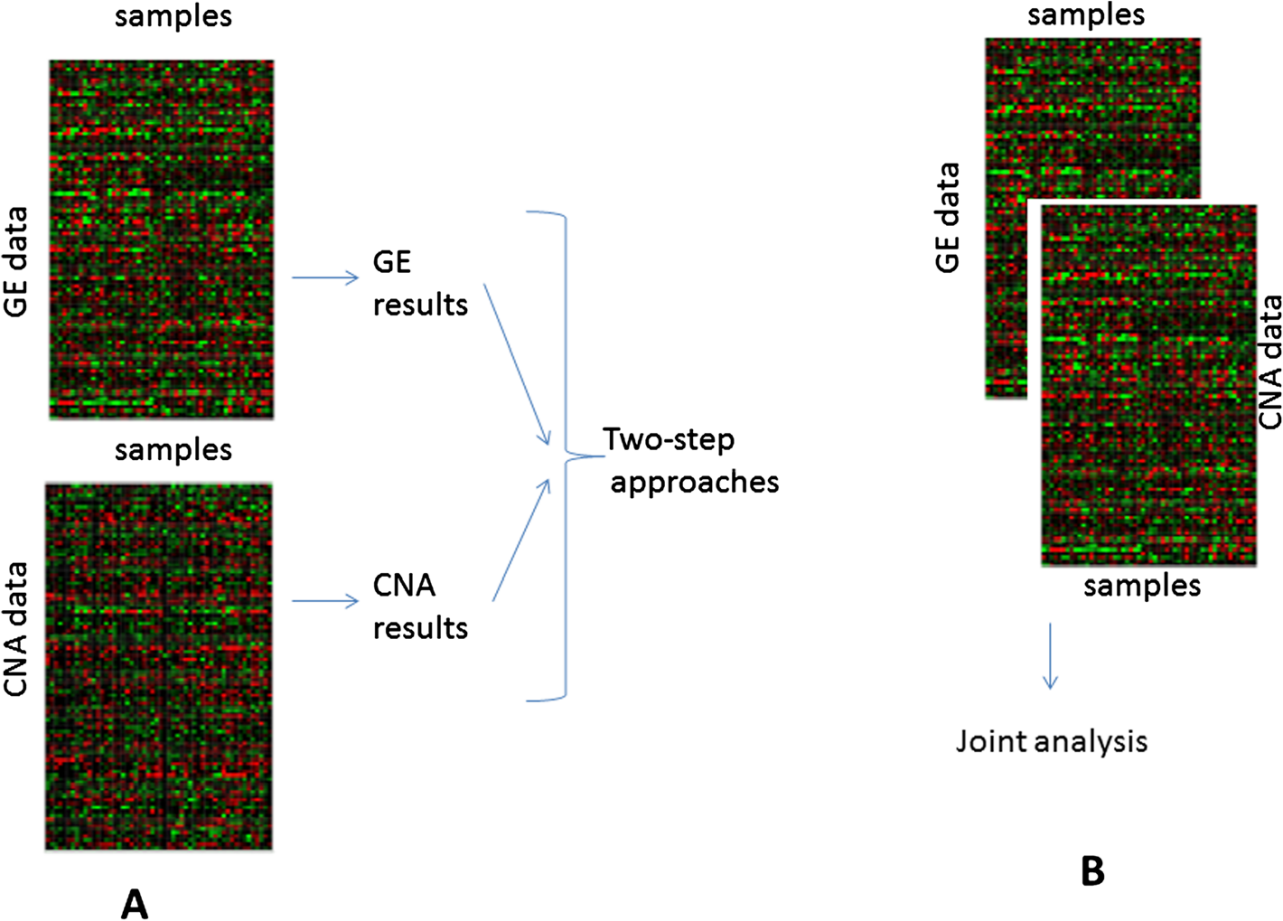
Integration approaches between GE and CNA data **a** two-step approaches, **b** joint analysis

Computational integrative methodologies between CNA and GE include a two-step approach, and joint analysis. Figure 5a) shows a two-step approach, combining the results from individual analysis of GE and CNA. Figure 5b shows a joint analysis obtaining directly the final result from the integration of GE and CNA.

There are different statistical measures to assess the CNA and GE relationship in order to quantify gene dosage effect. They include, in two-steps approaches, both regression and correlation-based analysis.

Regression approaches model the dependence of RNA levels from DNA CN, and consider RNA levels as responses and DNA CN as predictors [279]. These methods can be divided into: 1) univariate linear regression models, proposed to model the associations between individual CN and GE probes [280], 2) multivariate linear regression models, integrating statistical power across multiple probes targeting adjacent genes or chromosomal positions [281], and 3) nonlinear regression models.

Most studies use linear regression models but regulatory mechanisms, contributing to gene expression changes (e.g. CNA, miRNA, DNA methylation), can give non linearity [282]. Non linear relationship between CNA and GE have been investigated by Solvang et al. [282], which focused on the identification of nonlinear relationships to explain the regulatory mechanisms of alteration of mRNA expressions in the cancer process.

Correlation-based approaches have been used to study the relationship between CNAs and GE. For each pair of co-measured data, a correlation matrix was estimated reflecting the strength of association [283]. Several studies have shown correlations between CNA and GE gene across samples e.g. [277]. Other studies, like Tsafrir et al. [284], identified a correlation along the genome by using filtered CNA and filtered GE. DR-Correlate [285], a modified version of the Ortiz-Estevez algorithm, [286] was used in a correlation-based analysis to examine the genome and to detect genes with high associations between CNA and GE. In order to improved correlation results, Schäfer et al*.* [287] replaced the sample means with the reference medians in the correlation test, while Lipson et al*.* [288] used a quantile-based analysis to obtain improved correlation coefficients.

Table 8 reports a synthesis of the considered two-step analyses and types.

**Table 8:** Two step approaches to quantify gene dosage effect

Analysis	Type	References
- regression	• univariate linear	[279–282]
	• multivariate linear	
	• nonlinear	
- correlation	• signal-to-noise ratio	[283–288]
	• Pearson's correlation	
	• Algorithm Array CGH Expression integration tool (ACE-it)	
	• discriminating score	

Joint analysis uses CNA and GE data as paired data entries and not as separate structures. The discrepancy between the sample size and the number of genes is a problem that can cause high noise. Techniques such as Singular value decomposition (SVD) or Principal Component Analysis (PCA) are the most popular ones for reducing the dimension of gene data [289, 290]. However, GE and CNA data are separately analysed using these methods.

The generalized singular value decomposition (GSVD) is a popular regression framework used in joint analysis. With the purpose to identify variation patterns between two biological inputs, Berger et al. [291] applied an iterative procedure based on the GSVD, projecting CNA/GSE data into different decomposition directions. GSVD was used in two BC cell lines and tumour datasets, thus obtaining gene subsets that were biologically validated.

Soneson et al. [292] applied PCA to reduce dimensions, and Canonical Correlation Analysis (CCA) to identify highly correlated CNA/GE pairs. Gonzalez et al. [293] implemented the regularized CCA to identify the correlation between paired datasets. iCluster is a method able to generate a single integrated cluster assignment based on a simultaneous inference analysis from multiple data types [294]. In BC, iCluster has been used to align concordant DNA CNA and gene GE changes, showing encouraging results [294].

Table 9 reports some software for the CNA and GE analysis and their method of integration type.

**Table 9:** Software for CNA and GE analysis

Software	Integration type	References
Ace-it	two-step approaches	[295]
Magellan	two-step approaches	[296]
SODEGIR	two-step approaches	[297]
Edira	two-step approaches	[287]
CNAmet	two-step approaches	[298]
iCLUSTER	joint analysis	[294]
CONNEXIC	joint analysis	[299]
Remap	joint analysis	[281]
DR-Integrator	joint analysis	[285]

### Integrating genetics and epigenetics in BC

The development of BC is mediated by the cooperation, directly or indirectly, between genetic and epigenetic alterations of the cell [300, 301].

Sarkar et al. suggested that the epigenetic changes act as the initiating signal in the development of cancer progenitor cells and a combination of all genetic changes which are differentially expressed in the various cancer subtypes, could act on the cell vulnerable to epigenetic alterations [301].

Epigenetic mechanisms are tightly linked to one another and make the overall gene regulation system. The miR-29 family, for example, including miR-29a, miR-29b, and miR-29c, is a miRNA that collaborates with other epigenetic mechanisms. The expression of miR-29b is regulated by both histone modification [302] and DNA methylation [303]. miR-7/miR-218 can regulate DNA methylation and histone modification status by decreasing homeobox B3 (*HOXB3*) expression [304].

However, while classical epigenetic mechanisms, such as histone modification and DNA methylation, regulate expression at the transcriptional level, miRNAs act at the posttranscriptional level.

Elucidating the basic mechanisms of post-transcriptional regulation of GE is essential to gain a full understanding of how GE is regulated at different levels, of the interplay between these mechanisms, and of the extensive contribution of post-transcriptional dysfunction in cancer.

An impressive number of papers have been published on miRNAs increasing the number of scientific challenges, and we focused on the studies and methods applied to the combination miRNAs-mRNA, CNAs-miRNAs, and GE-genetic alterations-miRNAs.

### Integrated analysis of mRNA and miRNA in BC

#### Biological insights

The miRNA profile is more accurately associated with cell differentiation and cancer progression when compared with GE expression profile.

The aberrant expression of miRNAs in cancer can lead to the altered expression of target mRNAs. miRNAs can also modulate multiple genes regulating entire networks. The interaction of a miRNA with its target mRNAs can lead to the repression or incentive of GE.

In general, integration study of miRNA and mRNA may allow the identification of both biomarkers and networks involved in the development of cancer [305, 306].

##### Biomarkers

Combination of miRNA and mRNA has still to be deeply explored in diagnostic and prognostic studies. Cascione et al. [307] proposed a large-scale analysis of miRNA and cancer-focused mRNA expression in normal, triple negative tumour, and associated metastatic tissues in BC. Two miRNA signatures were identified, predictive of overall survival (*P* = 0.05) and distant-disease free survival (*P* = 0.009), respectively. Volinia et al. [308] found 30 mRNAs and 7 miRNAs associated with overall survival, across different clinical and molecular subclasses of BCs. In addition, expression profiles from 8 BC datasets, different from those used for the miRNA extraction, were used for validation. Buffa et al. [309] matched mRNA and miRNA global expression profiling, and four miRNAs were found independently associated with DRFS in ER-positive BC (3 novel and 1 known miRNA- miR-128a) and six miRNAs in ER-negative BC (5 novel and 1 known miRNA; miR-210). Van der Auwera et al. [310] identified a set of 13 miRNAs whose expression differed between inflammatory BC (IBC) and non-IBC. Enerly et al. [311] demonstrated, from the joint analysis of miRNA and mRNA data, a central role for miRNAs in regulating particular pathways. Hannafon et al. [312] identified putative miRNAs by mRNA functional interactions in ductal carcinoma *in situ*: the three *miRNAs miR-125b, miR-182* and *miR-183*, and six of their putative target genes, *MEMO1, NRIP1, CBX7, DOK4, NMT2*, and *EGR1*.

Luo et al. [313] performed an integrated analysis of miRNAs and mRNA expression profiles in 12 BC cell lines, identifying 35 functional target genes of three significantly down-regulated miRNAs in invasive cell lines (miR-200c, miR-205, and miR-375).

Several studies demonstrated the greater accuracy of miRNA expression levels compared with those of gene signatures. miRNA expression levels should directly represent the functional activity of the genes, while genes have to be translated to proteins to show their biological effects [314].

For a more detail review on the role of miRNAs and mRNA, see [315].

Table 10 reports a synthesis of the considered miRNAs biomarkers in BC as obtained by the integration of miRNA and mRNA.

**Table 10:** miRNA deregulated by integration miRNA and mRNA

Biomarker	Biological effect	References
*miR-16, 155, 125b, 374a*	predictive of overall survival (*P* = 0.05)	[307]
*miR-16, 125b, 374a, 374b, 421, 655, 497*	predictive of distant-disease free survival (*P* = 0.009)	[307]
*miR-1307, 103, 328, 83, 874, 484, 148b*	associated with overall survival across different clinical and molecular subclasses	[308]
*miR-767-3p, 128a, 769–3, 135a, and miR-27b, 144, 210, 42, 150, 30c*	associated with DRFS in estrogen receptor	[309]
*miR-335, 337-5p, 451, 486-3p, 520a-5p, 548d-5p, 15a, 24, 29a, 30b, 320, 342-5p, 342-3p*	associated with inflammatory breast cancer	[310]
*miR-150, 155, 142*	have strong positive correlation to the immune response module	[311]
*miR-130b, 19a, 449a, 299, 154, 145*	association with proliferation	[311]
*miR-29c*	associated with cell adhesion/extra cellular matrix	[311]
*miR-125b, miR-182 and miR-183*	highly overexpressed in ductal carcinoma *in situ*	[312]
*miR-200c, miR-205, and miR-375*	down-regulated miRNAs in invasive cell lines	[313]

##### Networks

Each miRNA can potentially regulate the expression of hundreds of genes, and a single gene can be targeted by multiple miRNAs [316].

Specific miRNAs has been identified as regulator of metastatic progression through miRNA regulatory networks. Yan et al. [317] found *miR-21* as the most significantly up-regulated miRNA in BC when compared with normal adjacent tumour tissues (NAT). Its target prediction revealed the putative target genes by creating a small miRNA regulatory networks.

**Fig. 6:**
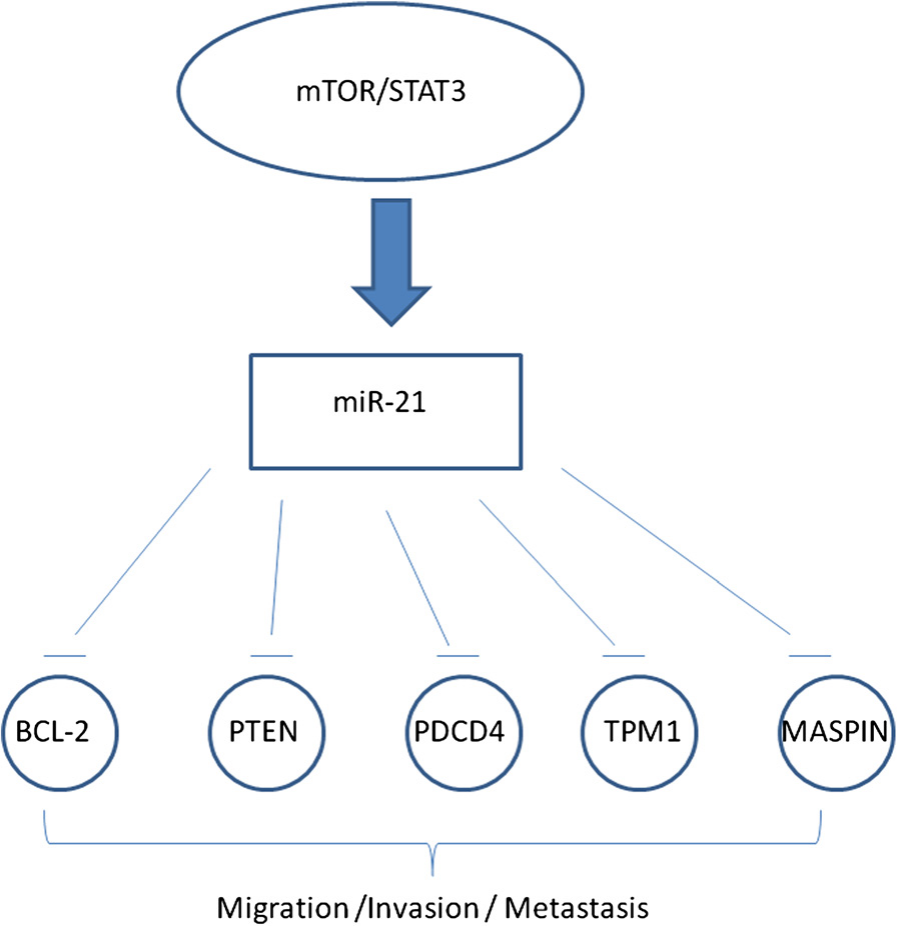
Example of miRNA regulatory networks

Figure 6 shows mTOR and STAT3 signalling acting on *miR-21* up-regulation in cancer [318] and *miR-21* promoting cancer cell invasion and metastasis through suppression of *BCL-2*, PTEN, PDCD4,TPM1, maspin [319]. The introduction of anti-*miR-21* to MCF-7 BC cells and in mouse model resulted in decreased cell growth (via increased apoptosis) and in reduced cell proliferation [319].

*miR-10b* was found highly expressed in BC metastatic cancer cells. *In vivo* studies demonstrated that *miR-10b* promotes cell migration and invasion [320, 321] and initiates tumour metastasis [320, 321]. *miR-10b* is induced by the transcription factor Twist. In turn, miR-10b inhibits HOXD10 and, through a cascade of cellular alterations, inhibits the expression of the prometastatic gene RHOC [320, 321].

*let-7* has been found poorly expressed or deleted in many cancers. Known oncogenic targets of *let-7* are *H-RAS, HMGA2*, and *BACH1*. These genes result down-regulated by *let-7* over-expression [322]. *HMGA2*, and *BACH1* promote the transcription of pro-invasive genes, suppress cell invasion and metastasis to the bone [323]. *let-7* is regulated by LIN-28, MEK signalling, and RKIP [323].

*miR-200* family is important in maintaining the tumour epithelial phenotype and in inhibiting the epithelial-to-mesenchymal transition (EMT). *miR-200* family was found to inhibit cell migration by acting on the transcription factors ZEB1 and ZEB2, which suppress E-cadherin [324]. Furthermore, *miR-200* was found silencing Sec-23a and promoting metastases by inhibiting *TINAGL1* and *IGFBP4* [325].

Table 11 reports a synthesis of the considered mRNA-miRNA networks.

**Table 11:** Example of network between miRNA and their targets in BC

miRNA	Targets	Phenotype	Ref.
*miR-21*	BCL-2,PTEN, PDCD4,TPM1, maspin	Migration, invasion	[318, 319]
*miR-335*	SOX4, Tenascin-C	Migration, invasion	[326–328]
*miR-10b*	HOXD10, RhoC	Migration, invasion	[320, 321]
*let-7*	H-RAS, HMGA2, LIN28, PEBP1	Proliferation, differentiation	[322, 323]
*miR-200*	BMI2, ZEB1, ZEB2, Sec-23a	Migration	[324]

#### Experimental methods

The most used experimental technique for determining miRNA targets is the transfection of mimic miRNAs or of miRNA inhibitors [329]. The consequences of the modulation of miRNAs on the expression levels are measured by using different tools, including RT-PCR or microarrays. The most important disadvantage of these techniques is that they are not able to discriminate between indirect and direct interactions [329]. Labelled miRNA pull-down (LAMP) assay system or luciferase report assays add reporters or labels to miRNAs on the 3'-UTR of transcripts of interest, allowing the identification and the analysis of direct interaction regions among miRNA and its target gene [7]. The disadvantage of reporter assays is that they are laborious, sensitive upon the region chosen for cloning, and that they require hard and complex work for trasfection [330].

#### Computational methods

There are different approaches to examine both miRNA and mRNA expression profiles. In this paragraph we examine miRNA and mRNA regulatory pairs together [183, 185, 187, 188]. Several studies showed that the miRNA-mRNA interactions varies with the development of disease [331, 332].

Recently, *in silico* studies used expression profiles to decrease the number of false positives and to enhance the number of biologically relevant targets [333, 334].

The integrative methods employ a three-step procedure: 1) Identification of DE miRNAs and mRNAs in the biological condition of interest. It can be done as reported in section 2 c); 2) Selection of putative miRNA-mRNA pairs (for instance, a prediction algorithm can be used to obtain the DE miRNA from DE mRNA. It can be done as reported in section 3 c); and 3) Identification of statistically significant miRNA-mRNA pairs. This last step needs the selection of an appropriate association measure, and the determination of its significance. The common assumption is based on the idea that regulatory relationship between any miRNA and its target mRNAs is an inverse correlation [335].

The mathematic tools consider simple correlation analyses (Pearson, Spearman) [336, 337], mutual information [337], linear regression [338–340], regularized least squares [341, 342] and bayesian inference [343, 344]. These methods give a score for each interaction mRNA-miRNA.

van Iterson et al. [305] used the global test [345] to associate each miRNA with the expression levels of a set of predicted mRNA targets. They suggest global tests to be better suited for integrated analysis of miRNA and mRNA expression data, compared with either Pearson correlation or lasso-based approaches.

Pearson Correlation is a measure of linear-dependency, widely used to show miRNA-mRNA showing a statistically significant correlation [346]. There are several web-tools that employ Pearson correlation for miRNA-mRNA target research (e.g. [349–353]).

Non-parametric (Spearman) correlation coefficient can be used as alternative measure of correlation. Usually it is chosen in case of outliers or with small number of measures. Contrary to Spearman correlations, Pearson coefficients require that both variables derive from a bi-variate normal distribution [348].

Mutual information is analogous to the Pearson Correlation but it is sensitive not just to linear dependencies, and can define whether two given variables are independent [348].

Multiple linear regression can evaluate the interactions between a set of miRNAs and a target mRNA, contrary to correlation measures which focuses on particular pairs interaction.

R-squared statistics is used for measuring the goodness of the fit of the data . When the number of samples with GE profiles is smaller than the number of covariates (e.g. miRNA), partial least squares can be applied [351]. This model gives those miRNAs explaining the maximum variance in GE profiles by ensuring a good fit of the model.

Lasso-based approaches are used to deal with undetermined linear system [342].

Bayesian inference use a priori information to estimate parameters and predict values in a probability framework. Several studies use this method for scoring putative miRNA-mRNA targets based on miRNA and mRNA expression data [352–354].

Table 12 reports a synthesis of methods considered for the integration analysis mRNA-miRNA.

**Table 12:** Methods for mRNA-miRNAs analysis

Method	Ref.
global test	[243, 282]
Pearson correlation	[346–350]
Spearman correlation	[348]
lasso-based approaches	[342]
Mutual information	[348]
Multiple linear regression	[338–340]
Partial least squares	[351]
Bayesian inference	[352–354]

#### Therapeutic approach

In the context of a network, miRNAs are able to regulate distinct biological cell processes like apoptosis, proliferation or receptor driven pathways, thus suggesting their possible use also as therapeutic targets or tools [147]. The most important advantage, with respect to other approaches targeting single genes, is their ability to target multiple molecules.

There are two main approaches to target miRNA expression in cancer. Direct approaches involve the use of oligonucleotides or virus‐based vectors to either block the expression of an oncogenic miRNA or to reintroduce a TS miRNA lost in cancer. Indirect approaches involve the use of drugs to modulate miRNA expression by targeting their transcription and their processing.

We think that the miRNAs described in the following sections could be interesting for the development of possible therapies in BC.

- Ma et al. [321] found *miR-10b* up-regulated in BC and explored a possible therapeutic application in an animal model of BC-bearing mice. The silencing of *miR-10b* with antagomiRs reduces miR-10b levels and increases *miR-10b* target, *HOXD10*. The therapy decreases metastases and was well tolerated by mice.
- Multiple studies have also shown a significant association between *miR-206* and *ER* in BC (e.g. [157]). In mouse models, the overexpression of *miR-206* was found significantly decreasing metastatic activity for 2 BC cell lines: BOM1 (highly metastatic to bone) and LM2 (highly metastatic to lung) [355].
- *miR-125* was found to be significantly down regulated in BC patients [356]. Experimentally, over-expression of miR-125 reduces ERBB2 and ERBB3 cell motility, and also reduces invasiveness of other numerous cancers [357, 358]
- *miR-34* is down regulated in BC cell lines and tissues, compared with normal cell lines and adjacent non-tumor tissues [359]. Expression of *miR-34* was found correlated with p53 status. In fact, silencing of p53 in human tumour cell lines decreases in *miR-34* level [360]. Moreover, as reported by Weidhaas et al. [361] *miR-34* levels change levels significantly after irradiation. A potential use for *miR-34* as radiosensitizing agent could be envisaged.
- *miR-155* is also linked to key cancer pathways as the gene is up-regulated by mutant p53 in BC, thus facilitating tumour cell invasion [362]. *miR-155* has also attracted considerable interest as a putative therapeutic target [363].

Table 13 reports a synthesis of the considered miRNAs, their potential target and function.

**Table 13:** Potential therapeutic miRNAs

miRNA	Potential Target	Function	References
*miR-10b*	RHOC	Invasion and metastasis	[321]
*miR-206*	ER	Metastasis	[155]
*miR-125*	ERBB2, ERBB3	Coordinate suppression	[356–358]
*miR-34*	*p53, CCND1*, *CDK4* and *CDK6,*	cell cycle	[359–361]
*miR-155*	*p53*	cell cycle	[362, 363]

### Integrated analysis of CNA and miRNA in BC

#### Biological insights

Many miRNAs are frequently located at fragile sites of the genome, which are usually either amplified or deleted in human cancer [364]. The aberrant miRNA expression in BC, in part, is due to these genomic alternations.

Zhang and colleagues studied 283 known human miRNAs in BC and showed that 72.8 % of miRNAs are located in regions that reveal CNAs [365]. In a recent study, miRNAs were shown to be up-regulated in gain regions compared to copy-neutral regions in BC, although the effect on miRNA expression was not incisive [366]. Iorio et al. [129] compared BC CGH data with independent miRNA expression by miRNA microarrays, and demonstrated that 81.8 % of miRNAs increased expression level and showed high DNA CN, and that 60 % of miRNAs exhibit decreased expression level with loss of DNA CN.

**Fig. 7:**
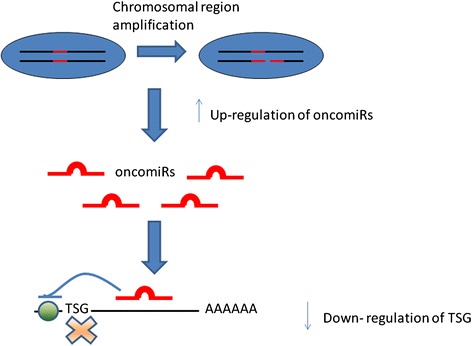
Amplifications of chromosomal regions of oncomiR lead to their up-regulation. These oncomiRs would then silence the TSG leading to the development of cancer

Several miRNAs have been associated with cancers due to CNA, suggesting that miRNAs can act either as oncomiRs or oncosuppressor miRNA [213]. Figure 7 shows amplification of chromosomal regions of miRNAs encoding oncomiRs and leading to their up-regulation. OncomiRs can act silencing TSG thus making possible the development of cancer.

##### Biomarkers

The first miRNA found to act as a mammalian oncogene is polycistron *miR-17-92*, also known as OncomiR-1 because it was the first identified oncomiR [367]. It is located in chromosome 13 and has been found amplified in human BC [368]. It acts as an anti-apoptotic miR cluster by targeting intrinsic apoptotic protein Bim in B-cell lymphoma subtypes [369].

Other oncomiRs have been described since the first discovery. *miR-21* is located in 3'UTR of VMP1 (vacuole membrane protein 1) gene at chromosome 17q23.2, a region amplified in BC and also in neuroblastomas, colon and lung cancers [370]. *miR-151a-5p* is located on 8q24.3, a genomic site frequently associated with gain in BC [371]. High expression of *miR-151a-5p* has been associated with gain, and functional experiments showed that over-expression induce cell proliferation and also increase the levels of p-AKT [372].

**Fig. 8:**
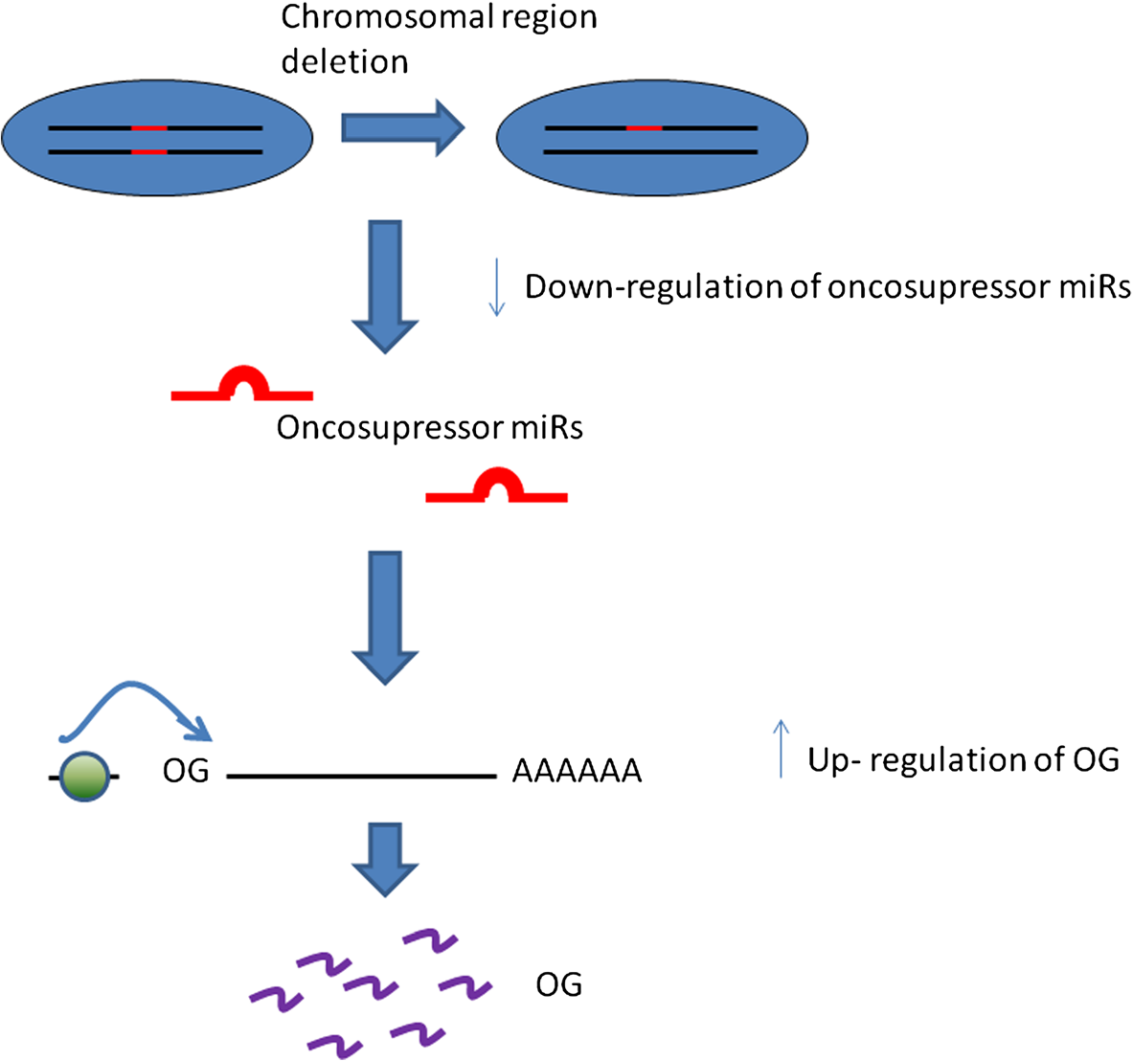
Deletions of chromosomal regions of oncosuppressor miRNAs lead to their down-regulation. Down-regulation of oncosuppressor miRNAs results in up-regulation of oncogenes and thus proliferation of cancer cells

As for oncomiRs, also several miRNAs with oncosuppressor functions have been described. Figure 8 shows deletion of chromosome region of oncosuppressor miRNAs leading to their down-regulation. Down-regulation of oncosuppressor miRNAs results in up-expression of target oncogenes.

Chromosome 11 is frequently altered in BC and *mirR-125b,* that is located at 11q23-24, results one of the most frequently deleted regions [373]. In a study of Muller et al. [374], *mir-320* has been found to be located in regions with DNA CN loss in BC. The predicted target of *miR-320* is *MECP2* which is up-regulated in BC and serves as an oncogene promoting cell proliferation. Genetic deletion could contribute to *miR-100* down-regulation [375] inducing epithelial-mesenchymal transition.

In several cancer types, including BC, genomic deletion or loss of heterozygosis of the region of the *miR-34a* have been described [376]. *miR-34a* is highly expressed in normal tissues. Its expression level is under the control of the TS gene product p53 and it acts as a TS inducing cell cycle arrest in G1-phase, senescence and apoptosis [377].

Wang et al. [378] showed that CN deletion is an important mechanism leading to the down-regulation of expression of specific *let-7* family members in BC. Also miR-33 expression was found to be strongly associated with the genomic alteration [128]. Furthermore, the expression of the cluster *miR-145/miR-143* family, miRNA located on a region involved in several types of translocations and deletions, has been found reduced or absent in various types of cancers, including BC [152, 368].

Table 14 shows the principal oncomiRs and oncogenes with their alterations considered in this section.

**Table 14:** miRNAs altered obtained by the integration miRNA and CNA

miRNA	Genetic alterations	Ref.
*miR-125b*	Deletions	[373]
*mir-320d*	Deletions	[374]
*let-7 g*	Deletions	[378]
*miR-34a*	Deletions	[376]
*miR-100*	Deletions	[375]
*miR-145*	Deletions	[112]
*miR-143*	Deletions	[112]
*OncomiR-1*	Amplifications	[367]
*miR-21*	Amplifications	[369]
*miR-155*	Amplifications	[368]
*miR-151a-5p*	Amplifications	[370, 371]

##### Networks

miRNAs that are silenced or amplified from CNA can have a cascade effect on the expression of different genes regulating entire pathways.

In the following paragraph, we give examples of important miRNAs that are altered in BC and of the consequences of their downregulation in the functional pathway.

**Fig. 9:**
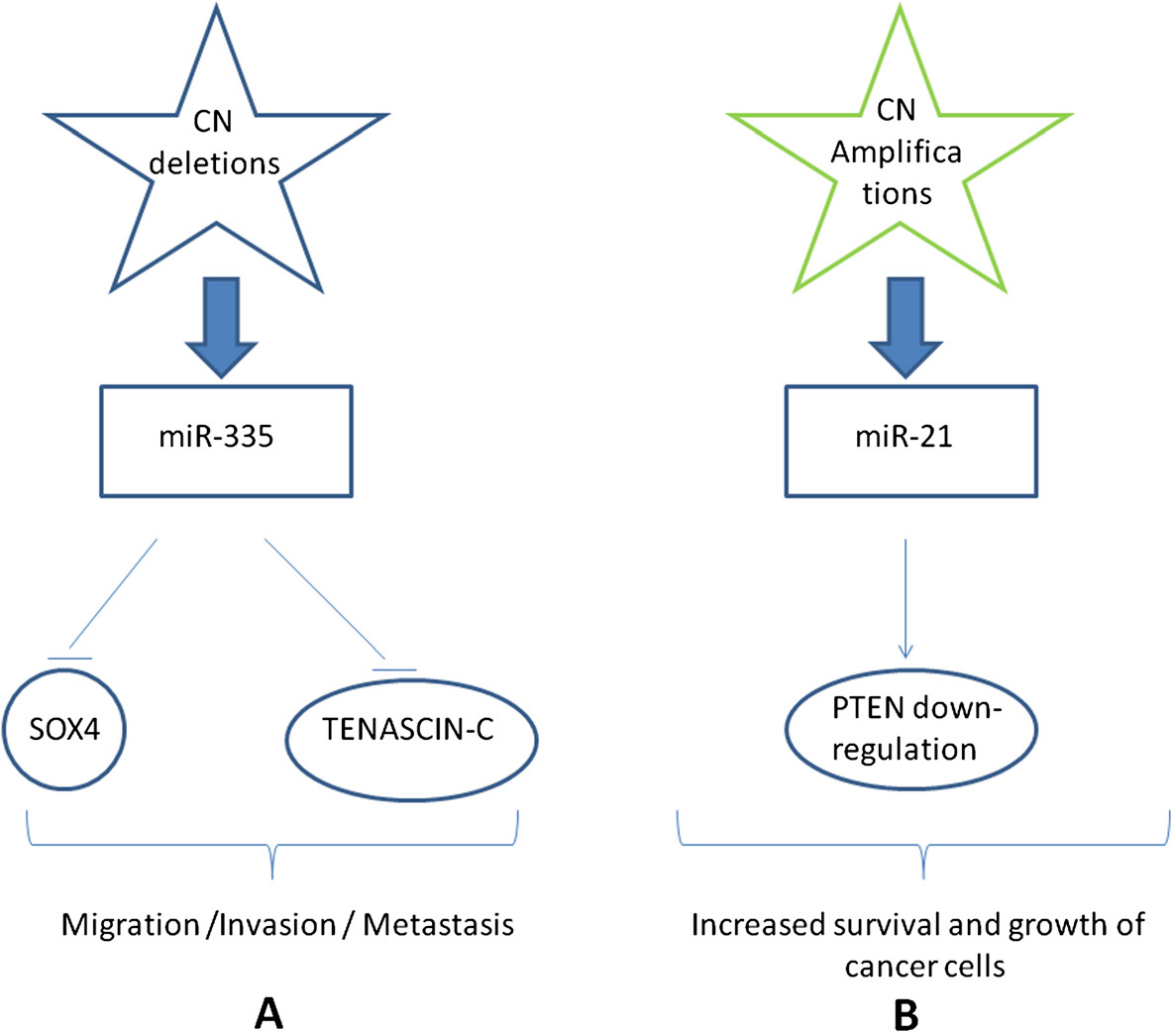
Examples of CNAs regulatory network. **a**) Deletions of *miR-335* produces effect that appear as promoting migration, invasion, and metastasis. In particular, it has been shown to be an important negative regulator of SOX4, and TENASCIN-C **b**) Amplifications of *miR-33* produce effects that appear as dyseregulation of PTEN pathway

Figure 9a shows *miR-335* that suppresses BC metastasis by targeting *SOX4* and *Tenascin-C* which promote cancer cell migration, invasion and ultimately metastasis [326–328]. *miR-335* is silenced through CN deletions [328].

*mir-320* is found to be located in regions with CN loss in BC. The predicted target of *miR-320* is methyl CpG-binding protein 2 (*MECP2*), which is up-regulated in BC and is an oncogene promoting cell proliferation [374].

In a study of Volinia *et al.* [368] *miR-21* was found as the only miRNA up-regulated in all six types of solid cancers (BC, colon, lung, prostate, stomach carcinomas and pancreas exocrine tumours). Figure 9b shows *miR-21* network: it modulates gemcitabine-induced apoptosis by *PTEN-*dependent activation of PI 3-kinase and by activation of AKT/mTOR signalling [379]. Inhibition of this miRNA should result in cell death [370].

#### Computational methods

Several studies showed that miRNA levels are influenced by CNAs.

No-experimental methods are usually used for their integration. Individual studies from miRNA and CNA are combined with statistically and/or computational analysis.

de rinaldis et al. [380] analysed association between miRNA expression and CNAs in a large triple-negative BC data set. This association was evaluated using Spearman correlation. In addition, for each miRNA-encoding DNA locus identified as altered in any of the samples, a separate non-parametric Wilcoxon rank sum test was applied to measure differences in expression between samples with deletions and amplifications, compared to samples with no CNAs. 64 miRNAs were found with statistically significant miRNA-CNA correlation, showing an overall influence of genetic alterations (amplifications and deletions) on the expression of the miRNAs.

Aure et al. [372] investigated individual and combined effects of CN and methylation on miRNA expression in BC. They identified 70 miRNAs whose expression was associated with CNAs or methylation, or with both conditions. 24 miRNAs were associated mainly with CNAs, 22 miRNAs with methylation aberrations and 24 miRNAs with a combination of CN and methylation aberrations. In order to identify miRNAs associated with hypomethylation or amplification, each miRNA in each patient was allocated to one of the two groups ‘altered’ or ‘non-altered’ based on CNA and DNA methylation. A Wilcoxon rank-sum test was used for each miRNA to underlie whether the miRNA expression was significantly different in the two groups.

Srivastava et al. [381] showed that *H2AX* was negatively correlated with *miR-24-2* and not in accordance with the CNA status, both in cell lines and in sporadic BC tissues. The authors tried to explain the possible mechanisms of such non concordant relationship between expression and number of gene copies based on specific miR regulation of expression. They discussed a role of miR-24-2 in guiding *H2AFX* GE in the background of the differential status of CNA.

### Combination of gene expression, genetic alterations and miRNAs in BC

Fearon and Vogelstein [382] proposed that accumulation of genetic alterations could determine a malignant phenotype and accompany cancer progression. However, this theory does not explain the great heterogeneity of observed genetic alterations, even within homogeneous histological groups [12].

Normal cells evolve progressively to a neoplastic state, based on a multistep process to acquire the traits that enable them to become tumorigenic and ultimately malignant. Tumors are not only masses of proliferating cancer cells, but complex tissues composed of multiple distinct molecular types that participate in an interaction with one another [383, 384].

The transitions in the malignant cancer progression are dynamic and reversible steps between multiple phenotypic states (e.g. epithelial and mesenchymal phenotype) [385]. These reversible transitions are based on complex epigenetic regulatory mechanisms (e.g. the induction of changes in the modifications of chromatin-associated histones) during epithelial-mesenchymal transitions [385, 386].

Sarkar et al. [387] reported a review based on the role of epigenetic regulation in the steps from normal cell to cancer progenitor cells that, after growing, undergo an epithelial-mesenchymal transition. Epigenetic drugs could potentiate traditional therapeutics by inhibiting both the formation and growth of cancer progenitor cells [387].

We argue that tumour heterogeneity is due not only to a simple accumulation of genetic alterations but can be the cause of the combined effect of genetic and epigenetic alterations. Furthermore, Alfred Knudson [388] hypothesized that hereditary retinoblastoma involves two mutations, the first one in the germ line. Thus, non-hereditary retinoblastoma should be due to two somatic mutations, an hypothesis known as Knudson “two-hit” hypothesis. The two-hit hypothesis proposes that loss of a single functional allele, which may potentially results in expression of a truncated or mutated product, is insufficient to involve cellular functions.

Several studies support the validity of the "two hit theory" in BC. Meric-Bernstam et al. [389] applied this hypothesis in BC, and suggested that the second hit does not need to be a point of mutation or somatic loss, but it may be the epigenetic silencing of a gene.

Konishi et al. [390] showed that cell lines carrying one mutant and one normal copy of *BRCA1* have a normal cell phenotype, and they are normal until the second allele is lost through somatic mutation or epigenetic silencing.

Genetic and epigenetic events are two complementary mechanisms that are involved in carcinogenesis. It is not clear at all how these mechanisms influence GE during tumorigenesis.

In BC, integration analysis of GE, genomic changes and miRNA expression was adopted in a limited number of studies (e.g. [397–399]). Eo et al. [391] proposed a pathway-based classification of BC which integrates data on DE genes, CNA and miRNA. Pathway information was incorporated in a condition-specific manner. A 215-gene signature was found from 327 tumours. By using an independent data set, this gene signature was validated.

Cancer Genome Atlas Network [392] analysed BC by genomic DNA CN arrays, DNA methylation, exome sequencing, messenger RNA arrays, microRNA sequencing and reverse-phase protein arrays. They found biomarkers for gene expression subtypes and the presence of four main BC classes.

Kristensen et al. [393] used an integrated approach to identify and classify BC according to the most deregulated pathways that provide the best predictive value with respect to prognosis, and identified key molecular and stromal signatures.

In a combined analysis of miRNA and mRNA expression data, Blenkiron et al. [128] found a number of miRNAs DE among molecular tumour subtypes. Furthermore, they found that changes in miRNA expression correlate with genomic loss or gain.

PARADIGM was tested using CN and mRNA expression data [394], as well as with the addition of methylation and miRNA expression data [393].

Cava et al. [5] assessed the potential of a new triple approach by integrating mRNA expression profile, CNAs, and miRNA expression levels to select a limited number of genomic BC biomarkers and to obtain a more accurate classification of BC grade.

CNAs have been demonstrated to be able also to identify genes DE between drug-sensitive and -resistant BC cells when integrated to GE and microRNA expression profiles.

Yamamoto et al. [395] focused on miRNAs and genes located on the genome-amplified and -deleted regions. These genes showed also an altered expression in GE profiles. The authors analysed MCF7 and a parental BC cell line drug-resistance MCF7-ADR. *miR-505* was identified as a tumour suppressor, whose genomic region was found to be deleted in doxorubicin-resistant cells. Furthermore, *miR-505* seems to be regulated by its predicted target *Akt3* (an anti-apoptotic gene), by mRNA profiling coupled with downstream validation studies.

## Discussion

Despite promising initial results about the possible clinical implications of GE profiling, a more recent source of concern has been that gene signatures derived from the various studies show little overlap and poor reproducibility. This can be explained, from one side, by the complexity of the human genome which provides that different genes can be indices of the same message with identical outcomes. From the other side, one explanation can be the use of different types of arrays (of different sample quality) and the different parameters considered for the data analysis. However, GE analysis measures mRNA expression, which, by the central dogma of molecular biology, results from the transcription of DNA. Specifically, GE analysis give information on DE genes among different conditions, but do not consider primary alterations of DNA from secondary effects of disease, such as, in the case of cancer, proliferation and differentiation state. On the other hand, studies of DNA CNA allow important indices to be derived as drivers of cancer. Therefore, integrating DNA and RNA data has been proposed to clarify genetic some regulatory relationships in cancer cells.

Since 2001, a new term "microRNA" was introduced into the scientific literature, challenging the central dogma of molecular biology. miRNAs are segments of RNA that are transcribed from DNA in a way similar to mRNA but they are not translated into proteins. In short, instead of producing a protein, miRNA can block mRNA directly. Evidences demonstrated that their deregulation is associated to several steps of cancer initiation and progression. However, we think that the association of miRNAs and their mRNA targets is a more favourable approach to study cell differentiation and cancer progression when compared with GE expression or miRNA profile alone. It is therefore of great concern for researchers to investigate how miRNA expression is linked to known BC markers. Several advantages can be envisaged by miRNA analysis: i) miRNAs are certainly more stable due to their small size when compared to long mRNAs [127], ii) miRNA expression levels can characterize the functional activity of the target gene while genes have to be translated to proteins to be biologically functional iii) miRNA-based therapeutics have the ability to target multiple genes.

Misregulation of genes with consequence disruption of the gene function is often induced by epigenetic and genetics events. The epigenetic silencing of one allele may act in concert with an inactivating genetic alteration in the opposite allele, thus resulting in a total allelic loss of the gene [7, 8]. From this viewpoint a gene subjected to a different possible alterations (such as CNAs and target of miRNAs) and that presents DE levels between two conditions is a "weak" point of DNA and could be a key element for cancer development. In our opinion each cancer should have a signature with the description of a specific set of alterations. Based on these observations, targeting specifically and simultaneously multiple pathways subjected to different alterations may confer a greater therapeutic efficacy.

We argue that useful information has been revealed by analysing GE profiles alone, CNA data alone but or miRNAs, however, in order to have complimentary information in gene characterization, an integrative analysis of CNA and GE data and miRNA is necessary.

However, integrative analyses have some limitations: the most fundamental challenge is dimensionally, considering that more levels in the analysis increase the computational time and the dimension of unknown parameters [396]. In addition, at every step, there are problems of compatibility of the data, such as normalization to the same scale, batch effects, and use of different platforms.

Large-scale integration is possible only for few projects worldwide, given the high cost for all analyses to be carried out simultaneously and on the entire data set.

In referring to current studies of genetic changes associated with BC, we focused in particular on the processes controlled by CNA. However, DNA changes include other genomic rearrangements, such as somatic point mutations.

The analysis of the genomes of 100 tumours revealed more than 7400 somatic point mutations in 21416 protein-coding genes [397]. These mutations affect many of the well-established cancer related genes, such as BRCA1, RB1, TP53, PTEN, AKT1, CDH1, GATA3, PI3KCA. These genes control apoptosis, proliferation and cell cycle, and transcription. Other somatic mutations affect genes involved in signal transduction (APC, KRAS, MAPK2K4, SMAD4, CASP8, CDKN1B…). Somatic mutation in three main genes (TP53, PI3KCA, and GATA3) shows more than 10 % incidence across all BC [397]. One of the most commonly mutated TSG in BC is *P53* [398]. It is localized to chromosome 17p13 and its inactivation is important also in other cancer diseases. Several studies have investigated the predictive power of *P53* for response to treatments and outcome of BC patients [399–401]. Bertheau et al. [401] reported that *P53* base-pair substitutions are highly linked to specific BC molecular subtypes, being found in 26 % of luminal tumours (17 % of luminal A, 41 % of luminal B), in 50 % of HER2 amplified tumours, and in 88 % of basal-like carcinomas. The type of mutations changes according to the tumour subtype. Basal-like tumours present higher frequency of deletions. Furthermore, the authors found that non inflammatory locally advanced BC with mutated *P53* has a higher rate of response to dose-dense doxorubicin–cyclophosphamide chemotherapy than *TP53*-WT tumours. As recently reported [402], *P53* is at the centre of the hallmarks of cancer, supporting genomic stability, exerting anti-angiogenic effects, controlling tumour inflammation and immune response, and repressing metastases. In BC, mutations in *BRCA1* and *BRCA2* result in protein truncations as consequence of small insertions, deletions or nonsense mutations. Although *BRCA1* and *BCRA2* mutations are hereditary, these genes would also be involved in the development of sporadic BC. Compared with normal breast epithelium, many BCs have shown low levels of the *BRCA1* mRNA [403, 404], while *BRCA2* has been found the target of frequent loss of heterozygosity (LOH) in BC [405, 406].

Other omics data could be further integrated for a more inclusive analysis. Considering that proteins translate effects of CNAs into the biological functions of the cell, further studies could integrate protein-protein interactions networks with gene-gene co-expression networks. For example, by dissecting the protein-protein interaction network into disjoint sub networks, van den Akkerb et al. [407] found sub-population of genes by using pair wise GE correlation measures. The obtained genes were consistently found across different studies.

Also the DNA methylation could be integrated in a pathway analysis and could be combined with other biological data. Andrews et al. [408] integrated results from CNAs, GE profiling and methylation to identify differentially regulated pathways between a highly metastatic BC cell line and low metastatic parental cell line. Validation experiments confirmed that hypermethylated genes correlated with decreased expression in the metastatic, compared to the parental cell line.

Results generated from whole-genome analyses have been submitted in The Cancer Genome Atlas (TCGA) database, which includes CNAs, DNA methylation and GE profiles [409, 410]. These data might be used for integrative analyses of results generated from a single technology platform [411].

## Conclusions

Integrating genetics and epigenetics in BC may offer a powerful approach for the identification of biomarkers with diagnostic, prognostic and therapeutic potential. The experimental and computational methods presented in this review can be used to guide researchers for these integration studies.

## Abbreviations

BC: Breast Cancer
CNA: Copy number alteration
GE: Gene expression
miRNA: microRNA
ER: Estrogen receptors
PR: Progesterone receptors
HER2: Human epidermal growth factor receptor-2
CN: Copy number
WT: Wild type
TSGs: Tumor suppressor genes
OGs: Oncogenes
LOH: Loss of heterozygosity
FISH: Fluorescence *in situ* hybridization technique
SNP: Single nucleotide polymorphism
NGS: Next generation sequencing
dChip: DNA-Chip analyzer
HMM: Hidden Markov model
CNAG: Copy number analyser for GeneChip arrays
RMA: Robust multichip average
*EGFR*: Epidermal growth factor receptor
DE: Differentially expressed
RT-PCR: Real-time quantitative reverse transcription PCR
